# Meta-analysis and moderator analysis of the seroprevalence of hepatitis E in South-Eastern Asia

**DOI:** 10.1038/s41598-023-37941-0

**Published:** 2023-07-23

**Authors:** Yakubu Egigogo Raji, Ooi Peck Toung, Niazlin Mohd Taib, Zamberi Bin Sekawi

**Affiliations:** 1 Department of Medical Microbiology Faculty of Medicine and Health Sciences, Universiti Putra Malaysia, Serdang, Malaysia; 2 Department of Clinical Microbiology, College of Health Sciences, Ibrahim Badamasi Babangida University, Lapai, Nigeria; 3 Department of Veterinary Clinical Studies Faculty of Veterinary Medicine, Universiti Putra Malaysia, Serdang, Malaysia

**Keywords:** Microbiology, Infectious diseases

## Abstract

By 2030, the World Health Organization wants to decrease viral hepatitis incidence and mortality by 90% and 65%, respectively. One of the agents responsible for the increased burden of viral hepatitis is the hepatitis E virus (HEV). This emerging pathogen is prevalent worldwide causing both acute and chronic infection. The rising risk profile of HEV has become a source of increased global public health concern. Despite this challenge, South-Eastern Asia (SEA), where many at-risk people are found, lacks uniform HEV prevalence data. Therefore, a meta-analysis was conducted to assess the overall prevalence of hepatitis E in SEA. Using R statistical software, a random effect model was used to estimate the logit-transformed prevalence. Moderator analyses were used to investigate the potential sources of variation. Thirty-two studies comprising 29,944 with 6806 anti-HEV antibody-positive individuals were evaluated. The overall HEV seroprevalence in SEA was 21% (95% confidence interval [CI]: 17–27) with high heterogeneity. At the country level, Laos has the highest prevalence estimate of 39% (CI: 16–69). Also, the studied population, year of publication, duration of sampling, and diagnostic method are significant HEV prevalence predictors accounting for 22.61% of the observed heterogeneity. The high HEV prevalence found in this study necessitates coordinated national and regional efforts to combat this emerging disease.

## Introduction

Globally, millions of people are infected with viral hepatitis yearly; a significant public health concern. The primary known aetiological agents of viral hepatitis are hepatitis A, B, C, D, and E viruses^1^. Some of these agents cause infections that may result in severe complications such as hepatocellular carcinoma or even death^2^. According to estimates from the World Health Organisation (WHO), viral hepatitis is responsible for the death of approximately 1.45 million people annually^1,2^. About 5–10% of those fatalities are attributed to hepatitis A and E, while the remaining are caused by hepatitis B and C^1,2^. Thus, hepatitis E virus (HEV) is now considered a significant contributor to the rising global burden of viral hepatitis.

Furthermore, HEV genotypes (HEV-1 to HEV-8) are being identified from diverse hosts^3^. Five of these genotypes (HEV-1 to HEV-4 and HEV-7) are known to be of human clinical importance. According to seroprevalence studies, a third of the world’s population is at risk of HEV infection^3^. Equally, annual new infections, including symptomatic cases, are increasing worldwide. Some infections also result in fatalities, with HEV accounting for 3.3% of all viral hepatitis mortalities globally^3^. Higher seroprevalence rates are recorded in developing countries than the developed nations^4^. However, epidemics are restricted to only developing regions, particularly in Africa and Asia^4^. The increasing global hepatitis E burden can be attributed to the multiple transmission routes of HEV. These transmission routes include water, food, and blood-borne as well as vertical and zoonotic^4^. Although most infections by HEV are usually mild and self-limiting^4^, certain groups are at risk of severe and or chronic disease. Acute HEV infection in pregnant women, mostly in developing countries, may present with fulminant hepatic failure with high mortality rates^4,5^. At the same time, chronic disease is seen in immunocompromised populations, mainly in developed regions^4^. Thus, HEV seriously threatens global health in developed and developing nations.

So, with the renewed effort by WHO to reduce the global burden of viral hepatitis^1^, providing a comprehensive information on the epidemiology of hepatitis E is critical. The need for this critical data is even more pertinent in the South-Eastern Asia (SEA) sub-region. This objective can be achieved efficiently through conducting a systematic review and meta-analysis. Thus, this study was undertaken as a follow-up to the previous systematic review (SR)^6^ by pooling the estimated effect of the seroprevalence of HEV from the included studies. Also, to investigate possible sources of heterogeneity through moderator analysis.

## Methods

### Study design

This study is an addition to an SR that was previously published^6^. The earlier review was conducted according to the PRISMA (S1 File) guidelines^7^. The review was preceded by developing an a priori protocol based on the PRISMA-P checklist^6^. Identified citations were screened based on some prespecified eligibility criteria (S2 File). Additionally, only studies adjudged to be of high quality after quality assessment were included in the previous review. Thus, all included articles in this study are of high quality.

Outcomes:

- To determine the overall seroprevalence of hepatitis E in south-Eastern Asia through meta-analysis.
- To determine factors that influence hepatitis E virus seroprevalence using moderator analyses.

### Analysis

#### Meta-analysis

The R studio desktop programme (version 2020.02.3 + 492) through the R software (version 4.1.2-2021-11-01) environment^8^ was used to conduct the meta-analysis. The pooled estimate prevalence (logit transformed) was obtained by adopting the random effect (RM) model. The generalised linear mixed-effect model (GLMM) method^9–11^ was used to pool the effect estimates. The confidence interval around the pooled effect was calculated using the Knapp–Hartung adjustment.

#### Assessment of heterogeneity

Heterogeneity was estimated by *X*^2^, Cochrane Q test, *I*^2^, and *T*^2^ statistics using restriction maximum likelihood (REML) estimation to calculate heterogeneity variance. The Cochrane Handbook of Systematic Reviews of Intervention^12^ guideline served as a reference for interpreting the heterogeneity threshold. Thus, an *I*^2^ value of 0–40% was considered not important; 30–60% represented moderate heterogeneity; 50–90% was regarded as substantial heterogeneity; 75–100% was deemed considerable heterogeneity. While a p-value of < 0.05 was interpreted as indicating significant heterogeneity. A 95% prediction interval (PI) was also estimated to determine the effect size variation across studies^13^.

#### Sensitivity analysis

Outlier and influence analyses were conducted to determine the robustness of pooled estimate and assess each study's influence on the summary proportion. Studies with extreme effect sizes were identified as outliers^14^ through the “find.outlier” function of “dmetar” package of R (version 4.1.2-2021-11-01) software. Influential studies that may push the effect estimate into one direction were investigated by conducting influence diagnostics using the “influential analyses” function of “dmetar” and “metafor” packages of R (version 4.1.2-2021-11-01) software. The influence diagnostics include the Baujat plot^15^, Viechtbauer–Cheung influence plot^14^, graphic display heterogeneity (GOSH) plot^16^, and leave-one-out meta-analysis.

#### Moderator analyses

Subgroup and meta-regression analyses were conducted to investigate the possible sources of the observed heterogeneity in the meta-analysis. Variables considered for evaluation as potential moderators were based on the reports from previous studies^17^. The variables used for the analyses include country of study, location setting (rural or urban setting), diagnostic method, assay type, and studied population. Others are gender, age, year of publication, sample size, and duration of sampling (in years). The age variable was determined for each of the included studies by the reported mean age. While gender was captured based on the predominant gender of the study population as reported in the study. The studied population variable was categorised as healthy, clinical, or mixed depending on whether a healthy general population, hospital-based (patients) or both respectively were sampled. For meta-regression, each of the covariates was assessed in univariate analysis. Only predictors with p < 0.25 were included in the multivariate meta-regression analysis model.

#### Publication bias

Potential publication bias was assessed by constructing and observing the funnel plot of the logit-transformed prevalence against standard error^18^. Egger’s regression test was further used to investigate the significance of the observed asymmetry^19^.

## Results

### Characteristics of included studies

**Figure 1:**
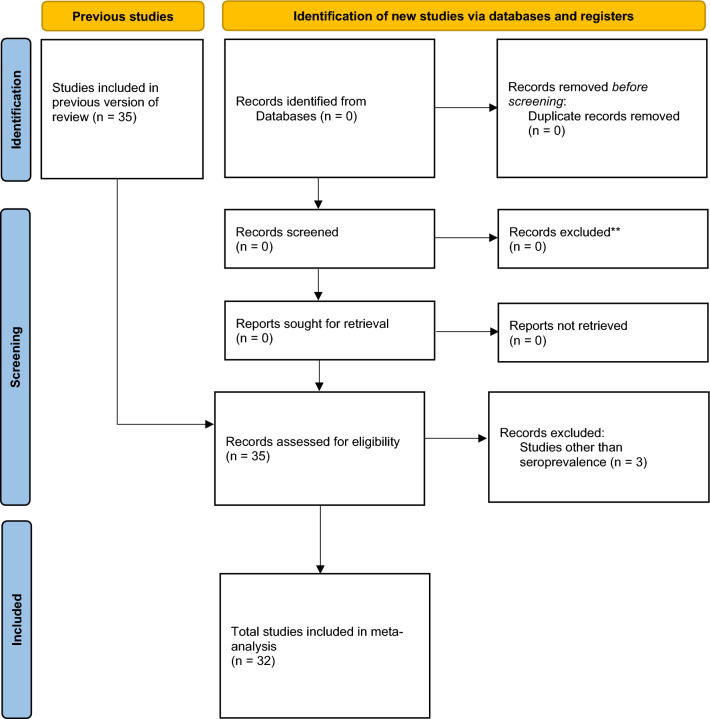
PRISMA flow diagram showing the screening process updated from the previous review.

All included studies are strictly seroprevalence studies evaluated from the previous review as high-quality studies^6^. Thirty-two studies from seven SEA states are included in the meta-analysis out of the 35 studies screened for eligibility (Fig. 1). The included studies with the quality assessment scores and the excluded studies are outlined in the S2 File. These studies comprised 29,944 individuals with 6806 positive for HEV antibodies. Out of the 32 included studies, 18 sampled the general (healthy) population, ten had clinical samples, and the remaining had a mixed population. The sampled general population has an overall sample size of 19,040 and 3622 positive events. Other relevant study characteristics are outlined in Table 1.

**Table 1:** Characteristics of studies included in the meta-analysis.

Study ID	Country	Duration of Sampling year	Sample size	Events	Location	Studied population	Mean Age	Gender
Nouhin et al., 2019	Cambodia	21	2004	824	Urban	Healthy	37	Male
Nouhin et al., 2016	Cambodia	1	301	85	Urban	Healthy	29	Male
Yamada et al., 2015	Cambodia	4	868	160	Rural	Healthy	31	Female
Nouhin et al., 2015	Cambodia	2	825	248	Urban	Clinical	29	Male
Wibawa et al., 2004	Indonesia	1	1115	73	Urban	Healthy	32	Male
Wibawa et al., 2004	Indonesia	1	797	144	Urban	Healthy	33	Male
Utsumi et al., 2011	Indonesia	1	253	25	Rural	Healthy	30	Female
Surya et al., 2005	Indonesia	1	819	151	Rural	Healthy	27	Female
Wibawa et al., 2007	Indonesia	1	57	23	Rural	Clinical	31	Male
Corwin et al., 1995	Indonesia	1	445	261	Rural	Mixed	30	Male
Widasari et al., 2013	Indonesia	1	490	38	Urban	Healthy	40	Male
Achwan et al., 2007	Indonesia	1	581	34	Urban	Healthy	50	Female
Tritz et al., 2018	Laos	1	326	169	Rural	Healthy	48	Female
Khounvisith et al., 2018	Laos	1	349	95	Urban	Healthy	32	Male
Hudu et al., 2018	Malaysia	2	82	8	Urban	Clinical	50	Male
Seow et al., 1999	Malaysia	1	232	62	Mixed	Healthy	20	Female
Ng et ai., 2000	Malaysia	1	145	21	Urban	Clinical	39	Male
Wong et al., 2019	Singapore	9	3261	727	Urban	Clinical	55	Male
Chow et al., 1996	Singapore	1	219	27	Urban	Clinical	52	Male
Sa-nguanmoo et al., 2015	Thailand	1	721	266	Urban	Healthy	32	Female
Pilakasiri et al., 2009	Thailand	1	381	44	Urban	Healthy	20	Female
Hinjoy et al., 2013	Thailand	2	513	118	Rural	Healthy	56	Female
Jupattanasin et al., 2019	Thailand	1	630	187	Mixed	Healthy	38	Male
Poovorawan et al., 1996	Thailand	2	900	61	Urban	Healthy	30	Female
Gonwong et al., 2014	Thailand	2	7760	1086	Rural	Blood d	21	Male
Siripanyaphinyo et al., 2014	Thailand	3	548	212	Urban	Clinical	49	Male
Hoan et al., 2019	Vietnam	2	451	202	Urban	Mixed	41	Female
Hoan et al., 2015	Vietnam	2	1658	691	Urban	Mixed	47	Male
Berto et al., 2018	Vietnam	5	2007	593	Urban	Mixed	40	Male
Hau et al., 1999	Vietnam	1	646	58	Rural	Clinical	21	Female
Tran et al., 2003	Vietnam	3	185	78	Urban	Clinical	21	Male
Corwin et al., 1996	Vietnam	2	375	35	Urban	Clinical	27	Male

### Meta-analysis

**Figure 2:**
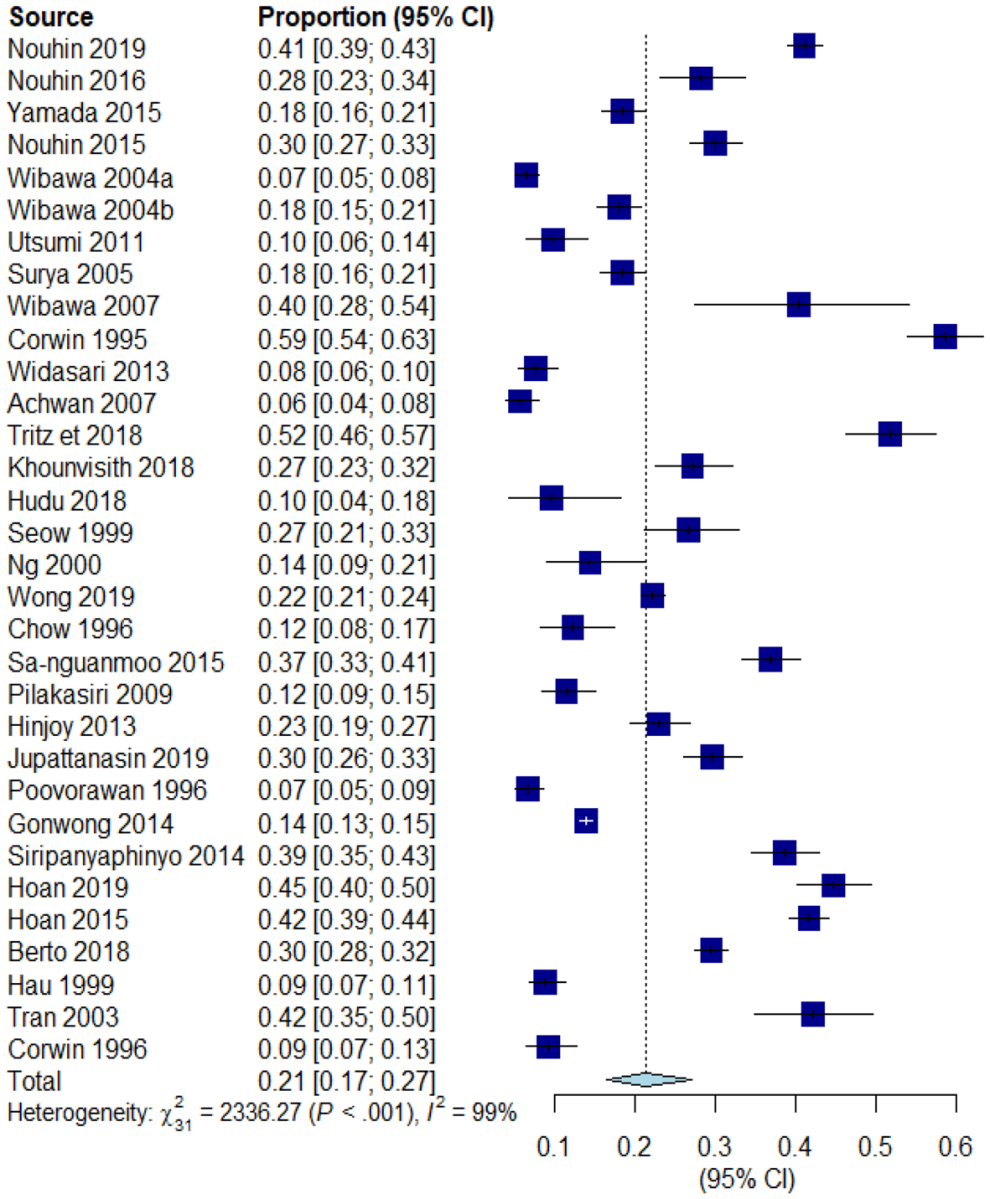
Forest plot presentation of the overall HEV infection seroprevalence meta-analysis.

The estimated HEV infection seroprevalence in the SEA region was 21% (95% confidence interval [CI]: 17–27; tau^2^ = 0.7341; tau = 0.8568; *I*^2^ = 98.7% [CI: 98.5%; 98.8%] Fig. 2). The estimated 95% PI shows that all comparable populations’ seroprevalence ranges from 4 to 64% (Supplementary Fig. 1).

### Sensitivity analyses

#### Outliers’ identification

The detected outliers were 19 studies^20–38^, and meta-analysis was recalculated using the remaining 13 studies^21,39–50^. The *I*^2^ has reduced from 98.7% to 88.9% from the rerun analysis, and the PI became narrower (S3 File: Item 2).

### Influence analyses

#### Baujat plot

**Figure 3:**
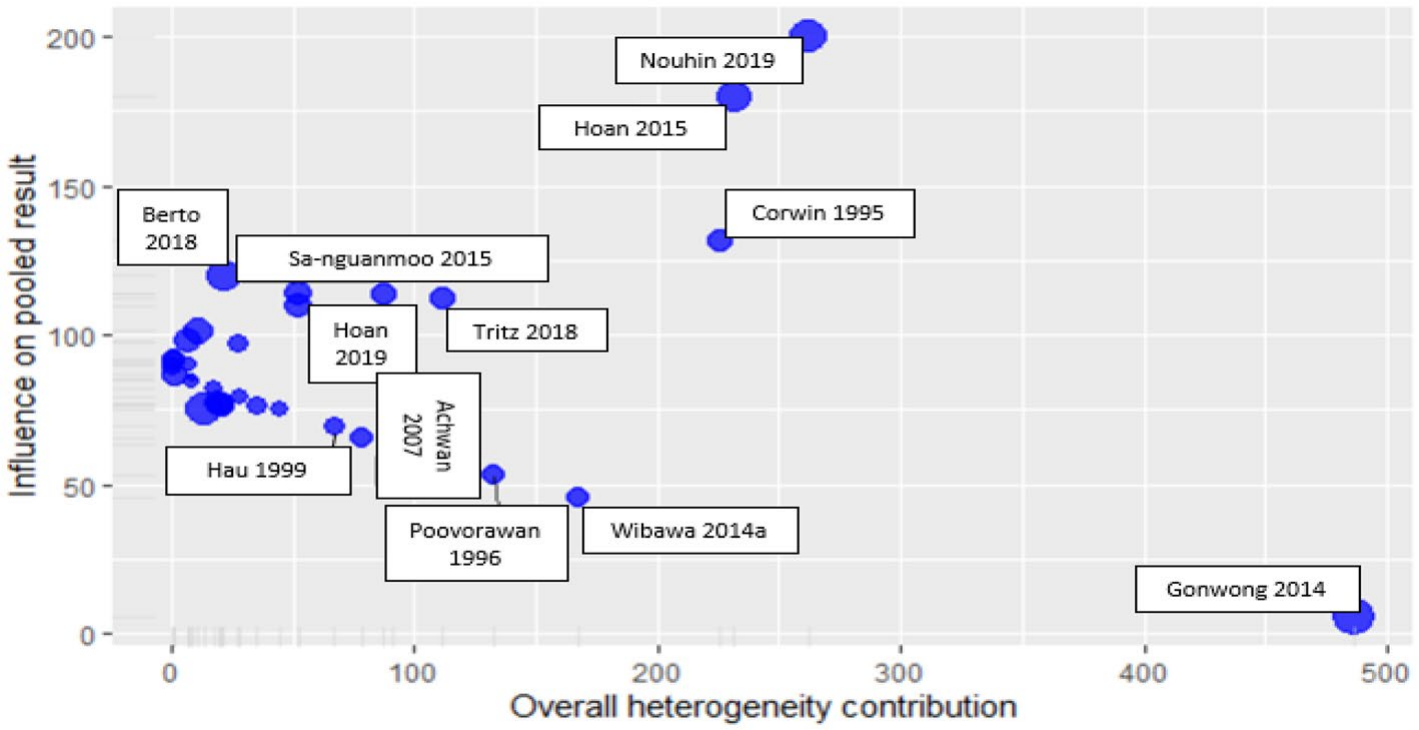
Baujat plot shows the influence of studies on heterogeneity and pooled effect.

The Baujat plot displays how each study contributed to the overall heterogeneity and how the studies affected the pooled effect. The studies^20,34,38^ in the upper right corner of the plot have a huge impact on both the heterogeneity and pooled effect, thus, are regarded as the most influential studies in the meta-analysis (Fig. 3). At the same time, the study^31^ in the lower right corner contributed heavily to the heterogeneity but not to the pooled effect.

### Viechtbauer–Cheung Influence analysis

From the spikes observed in the plot, the study determined to be influential is the Corwin (1995) study (Supplementary Fig. 2). This study was also identified in the two previous analyses.

### GOSH diagnostics

The plot revealed distinct effect size–heterogeneity clustering patterns of (1) high effect size–high heterogeneity (Supplementary Fig. 3) and (2) low effect size–high heterogeneity (Supplementary Fig. 4). This clustering pattern indicates more than one effect size population. Using the three clustering algorithms (S3 File: Item 4), the studies clustering in each of the clusters are outlined as follows:

- K-means clustering: Study 10, Study 1, and Study 13^20,24,27^.
- Connectivity (DBSCAN) clustering: Study 25, Study 1, Study 8, Study 10, Study 18, Study 23, Study 19, and Study 28^20,24,31,34,39,43,48,49^.
- Gaussian Mixture Model (GMM) clustering: Study 25, Study 1, Study 8, Study 10, Study 18, Study 23, Study 19, and Study 28^20,24,31,34,39,43,48,49^.

So, nine studies in total were identified by the GOSH diagnostics and the analysis was recalculated after removing the studies to determine their influence. After the removal of the studies, the *I*^2^ was 97.7%, and the Q statistic was still significant (S3 File: Item 5).

### Leave-one-out analysis

**Figure 4:**
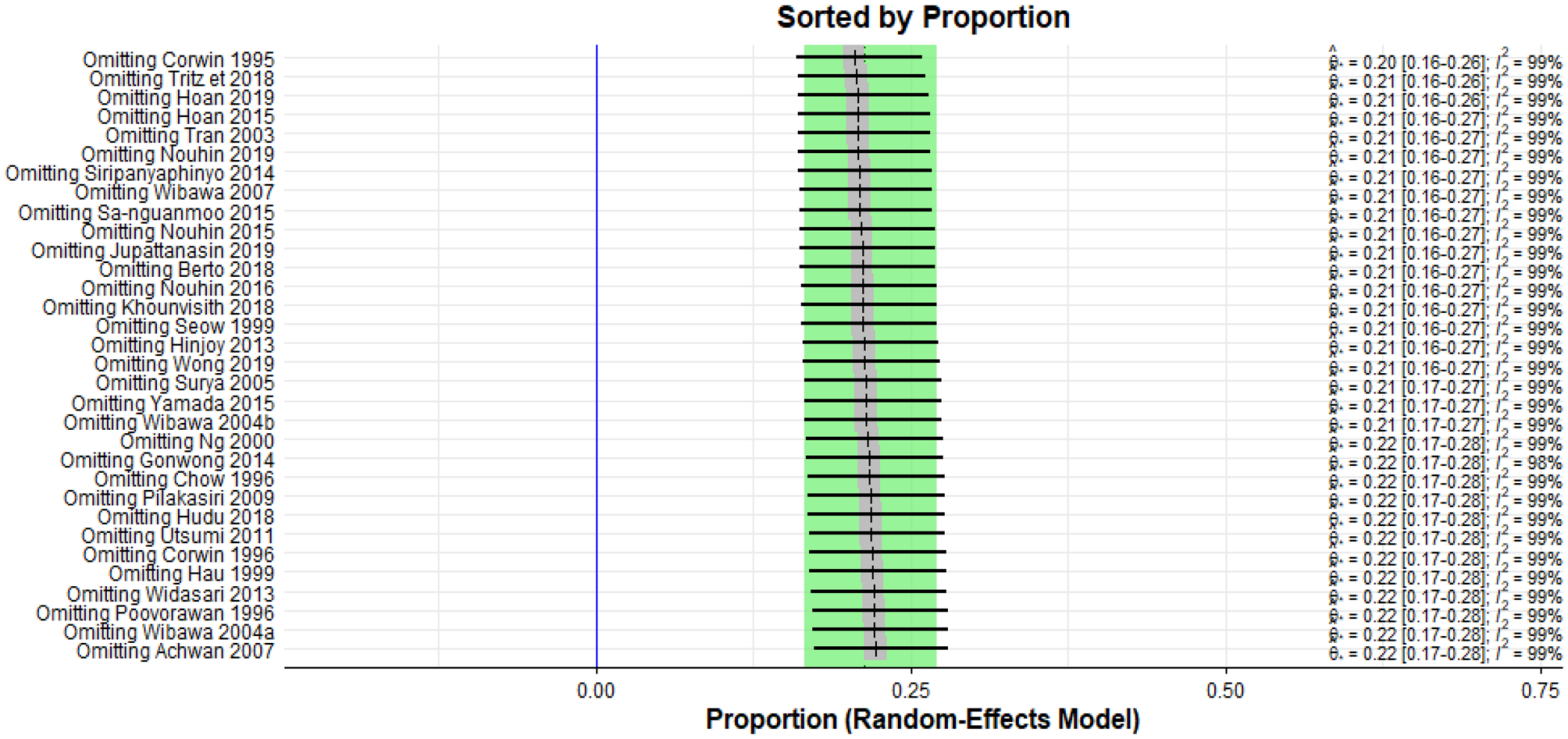
Graphical presentation of leave-one-out sensitivity analysis.

Sensitivity analysis was also done using the leave-one-out analysis to determine the studies that impact the robustness of the meta-analysis. Each included study was removed one after another, and the result was presented in a plot sorted by proportion (Fig. 4).

However, after conducting all the sensitivity analyses, the heterogeneity remained substantial, indicating that the identified studies could not effectively account for the observed heterogeneity. Therefore, moderator analyses must further explore the data's source(s) of heterogeneity.

### Moderator analyses

#### Subgroup analysis

**Figure 5:**
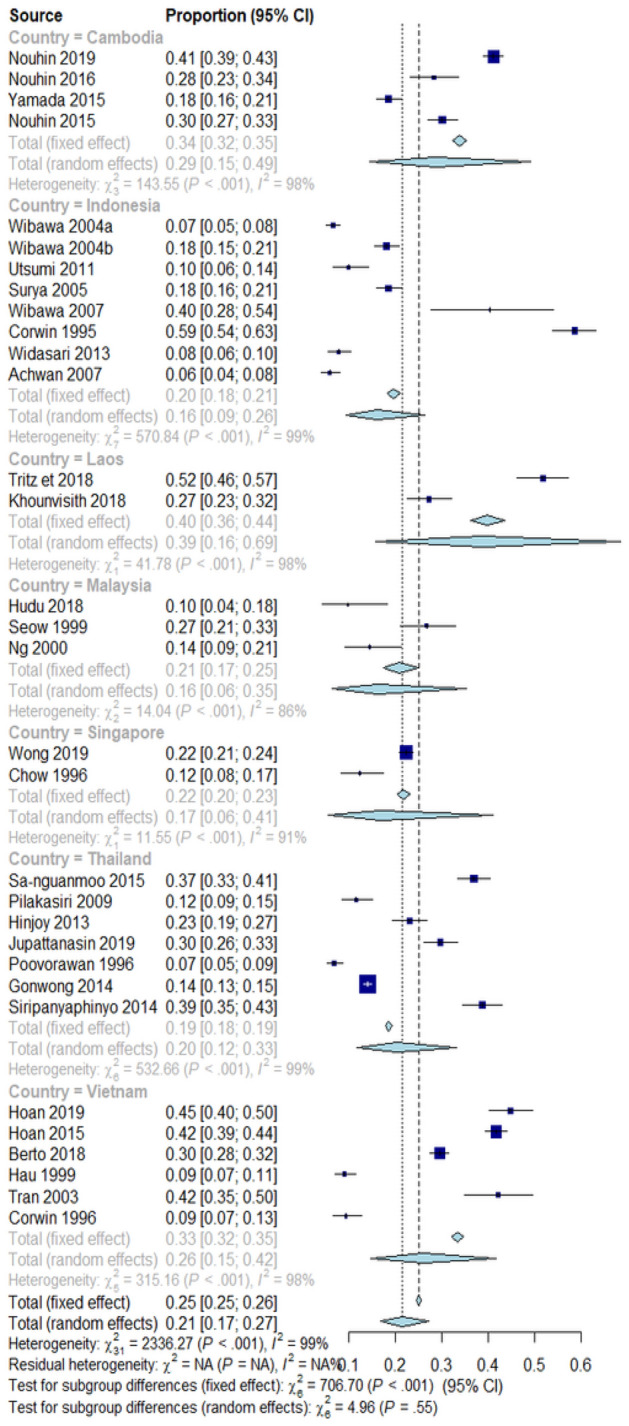
Forest plot for country-level subgroup analysis.

**Figure 6:**
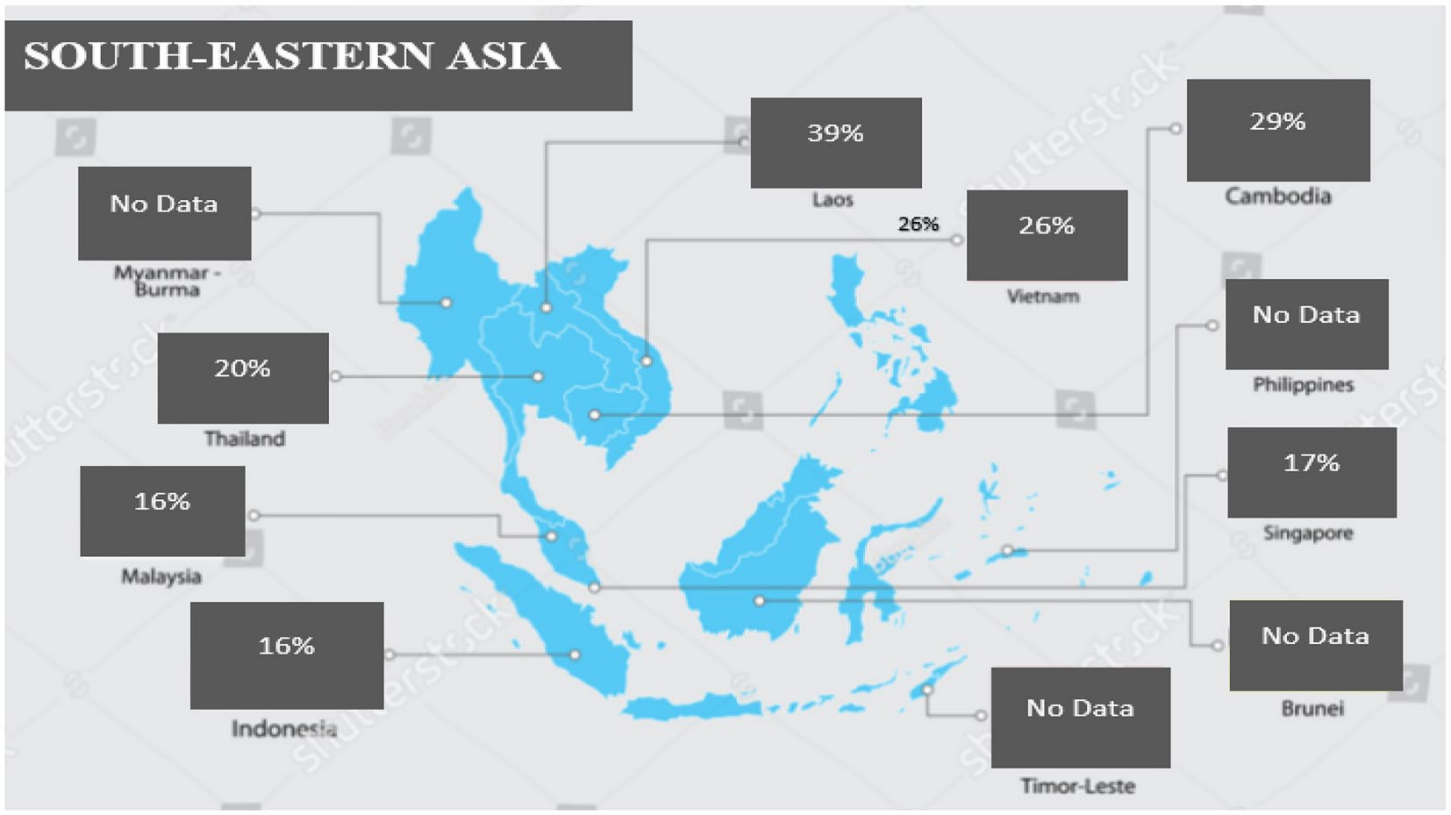
Map of South-Eastern Asia showing the respective countries pooled HEV seroprevalence.

Subgroup analysis based on the country of study revealed that among the seven evaluated countries in SEA, Laos People’s Democratic Republic (PDR) had the highest seroprevalence of 39% (CI: 16–69, tau^2^ = 0.2677; tau = 0.5174; *I*^2^ = 97.6%. Other countries' seroprevalences and their respective statistics are presented in the subgroup analysis forest plot (Fig. 5). A graphic presentation of the respective seroprevalence rates is shown in the SEA map (Fig. 6). Details of the subgroup analyses for the other evaluated variables are presented in Table 2.

**Table 2:** Subgroup analyses result in summary.

Variables	Number of studies	Pooled prevalence	Heterogeneity		*p* subgroup
		% (95% CI)	tau^2^	(*I*^2^)	
	32				
Country					
Laos PDR	2	39 (16–69)	0.2677	97.6%	0.0662
Cambodia	4	29 (15–49)	0.1588	97.9%	
Vietnam	6	26 (15–42)	0.7971	98.4%	
Thailand	7	20 (12–33)	0.5620	98.9%	
Singapore	2	17 (6–41)	0.1060	91.3%	
Indonesia	8	16 (9–26)	0.0966	98.8%	
Malaysia	3	16 (6–35)	0.2005	85.8%	
Location setting					
Urban	21	20 (14.3–27)	0.7456	98.4%	0.0456
Rural	9	23.7 (13.3–38.5)	0.8151	98.9%	
Mixed	2	29 (13.5–51.4)	0.0000	0.0%	
Diagnostic method					
Total Ig	11	27 (17.5–39.1)	0.6583	97.7%	0.1350
IgG	21	18.9 (13.7–25.4)	0.6935	98.5%	
Assay type					
Euroimmum, Lubeck	3	31.4 (22.1–42.5)	0.0275	84.6%	< 0.0001
Wantai Bio-Pharm	5	27.4 (16.4–42.2)	0.2504	95.6%	
MP Biomedicals	4	25.7 (7.6–59.2)	0.8013	99.1%	
DIA.PRO Diagnostic	2	24.2 (0.7–99)	0.4544	99.5%	
Genelabs Diagnostics	3	19.6 (0.9–85.6)	1.6305	99.4%	
Abbott laboratories	3	12.4 (2.6–28.5)	0.0000	0.0%	
Mizuo et al., method	4	17.6 (5.8–42.5)	0.5822	96.9%	
In-house assay	2	17.5 (0–99)	1.5030	99.1%	
WRAIR EIA	2	16.7 (0.5–89.4)	0.1549	94.6%	
Others	4	24 (7.8–54.2)	0.6695	98.1%	
Studied population					
Health	18	18.4 (13–25.3)	0.6596	98.8%	< 0.0001
Clinical	10	20.2 (12.6–30.7)	0.5815	96.6%	
Mixed	4	43.2 (27.7–60.3)	0.1820	98.0%	
Gender					
Male	20	23.3 (17.1–30.9)	0.6599	98.9%	0.3609
Female	12	18.5 (11.3–28.7)	0.8015	98.3%	
Age					
20–25	5	18.2 (8.2–35.5)	0.5178	97.2%	< 0.0001
26–31	9	21 (11.6–34.9)	0.8124	98.4%	
32–37	5	23 (9.7–45.5)	0.6748	99.0%	
38–43	4	18.5 (7.1–40.4)	0.4495	96.9%	
44–49	4	43.9 (36.8–51.2)	0.0267	81.4%	
≥ 50	5	13.4 (6.6–25.3)	0.3539	95.4%	
Year of publication					
≤ 1999	6	16.2 (6.1–36.5)	1.0504	99.0%	0.0198
2000–2004	4	17.2 (5.1–44.4)	0.6906	98.0%	
2005–2009	4	15.5 (4.5–41.5)	0.6888	95.8%	
2010–2014	5	16.4 (7.4–32.7)	0.5154	98.5%	
2015–2019	13	30.9 (24.6–38)	0.2581	97.5%	
Duration of sampling					
1–4 years	29	20.5 (15.5–26.6)	0.7737	98.6%	< 0.0001
5–9 years	2	25.7 (5.9–65.6)	0.0339	97.1%	
≥ 10 years	1	41.1 (39–43.3)	–	–	
Sample size					
< 100	2	21.4 (0.1–99)	0.7691	93.7%	0.8521
100–500	13	23 (14.4–34.6)	0.8644	97.9%	
> 500–1000	11	18.8 (12.2–27.9)	0.5719	97.9%	
> 1000–2000	2	18.3 (0.1–99)	1.3468	99.7%	
≥ 2000	4	25.5 (12.8–44.4)	0.2822	99.6%	

### Meta-regression analysis

#### Univariate analysis

Univariable meta-regression was used to ascertain the impact of study-level covariates on the pooled seroprevalence. The analysis revealed that the studied population, year of publication, duration of sampling, and diagnostic method are significant predictors. Table 3 outlines the respective proportions of the effect of the evaluated covariates.

**Table 3:** Meta-regression analyses.

Univariate analysis			Multivariate analysis		
Covariates	*R*^2^ (%)	*p* value	Covariates	*R*^2^ (%)	*p* value
Country	0.00	0.5490	Diagnostic method	22.61	0.0193
Location setting	0.00	0.6057	Studied population		
Diagnostic method	4.01	0.1483	Year of publication		
Assay type	0.00	0.9139	Duration of sampling		
Studied population	16.22	0.0206			
Gender	0.00	0.3644			
Age	0.00	0.6459			
Year of publication	10.17	0.0394			
Duration of sampling	1.98	0.2020			
Sample size	0.00	0.7715			

*R*^*2*^: explains the proportion of between study variance (the effect of covariates on heterogeneity).

#### Multivariate analysis

The three significant moderators in the univariate analysis were included in the multivariable analysis, accounting for 22.61% of the observed heterogeneity (Table 3).

#### Publication bias

**Figure 7:**
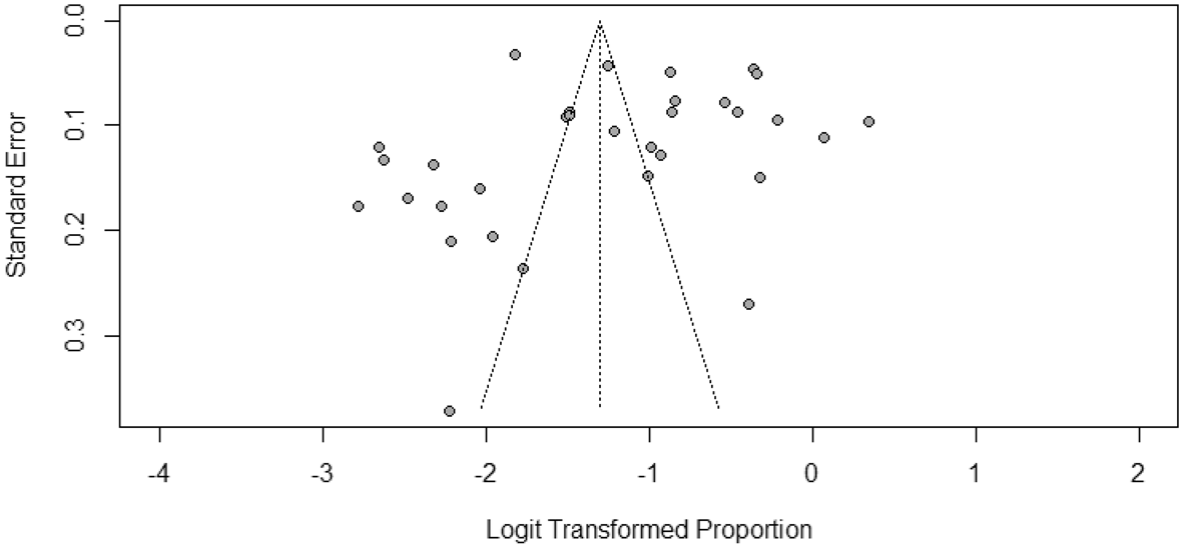
Funnel plot of logit-transformed prevalence against standard error showing observed asymmetry.

There is an observed asymmetry of the funnel plot, which illustrates potential publication bias (Fig. 7). Further quantitative evaluation of the observed asymmetry shows that Eggers' test does not indicate the presence of funnel plot asymmetry (Table 4).

**Table 4:** Quantification of funnel plot asymmetry.

Test	Intercept	Confidence interval	t	p
Egger’s regression test	− 2.025	− 7.89 to 3.84	− 0.677	0.5038

## Discussion

A comprehensive evaluation of HEV seroprevalence in SEA will assist in reducing the global burden of viral hepatitis as envisioned by the global health strategy on viral hepatitis^1^. The evaluation of HEV seroprevalence will provide the necessary information for informed decision-making concerning policymakers, public health practitioners and physicians. An informed decision based on a proper understanding of the disease epidemiology will help prevent and control hepatitis E. Therefore, this study provides data on evaluating the overall seroprevalence of hepatitis E and its associated predictors in SEA.

It has been established in an earlier review that hepatitis E is endemic in SEA, with evidence of its existence dating back more than 25 years^6^. Thus, it is unsurprising that the average prevalence of hepatitis E in this sub-region is high. The estimated seroprevalence in this study is higher than the global average of 12.5%^51^. This study's prevalence estimate is higher than the findings from some regions. Hepatitis E seroprevalence was estimated at 7.7% and 9.31% in the Americas and Europe, respectively^51,52^. The prevalence was 11.81% in the Middle East and 12.17% in the Eastern Mediterranean^53^. However, the finding of this study is similar to the average prevalence of 21.76% obtained in Africa^51^. In addition, this survey has also shown the possibility of even higher rates of up to 64% in future studies, as evidenced by the estimated prediction interval.

Different factors could be responsible for the observed high prevalence in SEA and the disparity from other regions. Apart from Singapore, a high-income country, the remaining countries with studies included in this meta-analysis are either low, lower-middle or upper-middle income^54^. Thus, the prevailing challenges of poor hygiene and environmental conditions associated with developing countries could have contributed to the high seropositivity rate. Numerous studies have already established that factors such as insufficient access to safe drinking water, floods, and inadequate health education are risk factors for HEV infection^4^. Additionally, contact with domestic and wild animals is not uncommon in this sub-region^4,51,52^. There is also the risk associated with consuming raw or undercooked animal products such as pork liver^4,52^. The unprecedented expansion in the livestock sector witnessed in this sub-region in recent years, and its attendant negative consequences may have aggravated these risk factors^55^. Some of these adverse effects include livestock-associated water and soil contamination and the threat of zoonotic disease surges^55^. Also, the SEA is a culturally and ethnically diverse region and experiencing a rapid increase in international tourism in recent years^56,57^. These two factors could also play a role in the high rate of HEV seroprevalence in the region. Another possible explanation for the high prevalence rate may be that some of the studies included in this meta-analysis used total antibodies for diagnosis. This method contrasts several other analyses that included studies that used only IgG diagnostic method.

Further, the high seroprevalence estimate was accompanied by substantial in-between study heterogeneity. It was discovered in this study that seroprevalence varied between countries. The seropositivity rates are high in all the countries, with Laos PDR identified as the country with the highest prevalence.

However, it should be noted that Laos PDR had only two studies included in the meta-analysis. Yet still, environmental and hygiene challenges peculiar to developing countries might be contributing factors. Others include increased contact with animals due to expansion in livestock farming. Livestock farming in Laos PDR is still largely traditional, with more of small-scale (backyard) local production system^58,59^. This farming system promotes more animal contact and the adverse effects associated with livestock farming that may serve as risk factors for HEV infection. There is equally the cultural dietary preference for consuming raw or undercooked animal products practised in many countries in SEA^60^.

Living in rural areas has been established in numerous studies as a risk factor for HEV infection. Thus, expectedly, higher hepatitis E prevalence were discovered in the rural than the urban areas. Other associated factors might have contributed to the observed outcome besides the earlier enumerated risk factors. The populace may have lower educational and economic status in rural areas than the urban settlers^61^. However, studies that combined urban and rural populations produced the highest prevalence. This phenomenon can also be explained by the increase in rural–urban migration and the possibility of sampling high-density urban areas^61^.

Similarly, this study shows that reported seroprevalence rates were impacted by the employed diagnostic method and assay type. Studies using total HEV antibodies as diagnostic methods showed higher prevalence than those using only IgG. Also, there was observed variation in HEV prevalence among the evaluated assays. The performance of assay types is often influenced by their specificity and sensitivity levels. HEV assay performance comparative studies have shown varied seroprevalence rates among the assays evaluated^62^. Thus, assays have predictive and modifier effects on HEV seroprevalence^17^. Therefore, the findings of this meta-analysis agree with several primary studies and meta-analyses^17,51,63^. The result did not show any specific pattern in sample sizes, probably due to the relatively small number of studies used in most groupings.

Furthermore, this current study also showed that the HEV seropositivity rate is lower in the healthy population compared to the clinical and mixed populations. The result further established that those with underlying diseases and the immunocompromised have an increased risk of HEV infection compared to the healthy population. Differences were also observed between genders, ages, years of publication, and sampling duration. The prevalence was higher in males than females, as previously established. Studies have reported higher HEV seroprevalence in males than non-pregnant females^4^. The most suggested reason for the propensity is the increased behavioural exposure in men compared to women^4^. This study also revealed a pattern that suggests an association of anti-HEV antibody positivity with increasing age. Prevalence was lowest in the age group of 20–25 years and peaked at 44–49 years age group. The observed pattern conforms with many primary and secondary studies^4,52^. Some explanations for this pattern include lifetime cumulative exposure and the impact of ageing on immunological function^52^. Others are genomic instability and other ageing processes; thus, prevalence tends to increase with age^52^.

Evaluation based on the year of publication showed almost a two-fold increase in HEV seroprevalence rate in 2019 from the 1999 rate. Likewise, it was observed that the longer the duration of sampling, the higher the prevalence. Studies with longer sampling duration had older samples indicating that studies with more recent samples had lower prevalence. This increase could be attributed to a substantial rise in HEV infection, increased awareness and research, or the use of more sensitive diagnostic assays. Although there are concerns that hepatitis E is still underestimated, recent discoveries about the epidemiology of the disease might have contributed to the observed rise in its prevalence as detected. These discoveries have led to a series of epidemiologic and pathological studies aimed at understanding the nature of the disease. For example, studies have shown constant expansion of HEV’s host range with an increased probability of cross-species infections^64^. This phenomenon may result in more human infection due to frequent contact with these animal hosts of the pathogen. Also, in recent years, there seems to be an increased awareness among researchers about HEV being a critical causative agent of viral hepatitis.

Additionally, most recent assay types have shown improved efficacy in diagnosing the disease over the old^4,65^. The use of more effective diagnostic assays will be more accurate compared to the less sensitive techniques. These factors, individually or in combination with the contribution of others, might have been responsible for the observed rate rise reported in this study. However, with increased awareness, clinical evaluation, and research, the prevalence might increase, as indicated by the prediction interval estimate.

On a more technical note, it was observed that substantial variation persisted in each of the assessed groups after subgroup analyses. Although heterogeneity is expected in the pooled effect size, it is assumed that the variation should be reduced significantly in close groups. Thus, its persistence in homogeneous subgroups calls for deeper exploration. So, further assessment using meta-regression analysis showed four factors (individually and in combination) that are significant moderators of the effect size. The quantifiable factors are studied population, diagnostic method, year of publication, and sampling duration.

Nevertheless, the predictors could only account for a fraction of the variation. Other factors that can potentially bias effect size estimates, such as file drawer effect, p-hacking strategies and publication bias, may equally affect the heterogeneity estimate^66^. However, the assessment showed that publication bias was not present in this survey. Though, as already established, heterogeneity could be due to quantifiable, hidden, or random moderators^66^. Other hidden factors that were not evaluated such as HEV genotype variation, might be responsible for the residual heterogeneity. Some others might even be entirely unknowable thus, cannot be specified or controlled^66^. So, pooled studies in meta-analyses can vary due to reasons that will likely never be fully identified^66^.

Consequently, primary prevalence studies must be designed as similarly as feasible to reduce between-study variation. The deduced recommendation from the findings of this study is for future seroprevalence studies to sample homogenous populations. Subsequent surveys at either national or regional levels can adopt a unified diagnostic method for hepatitis E investigation. In addition, as recommended by different researchers, further primary studies should consider regional and national surveys using similar study designs and analytical methods^66,67^.

As the first meta-analysis of hepatitis E seroprevalence in SEA, this study has pooled many studies, leading to a relatively large sample size with enhanced statistical power. Also, this experiment was conducted using meticulous methods, and moderators were thoroughly investigated. Therefore, a comprehensive sub-regional as well as national data on the seroprevalence of HEV is provided. The provided information will thus assist further research and informed decision-making for designing HEV preventive and control measures in the sub-region. However, there are a few limitations of this study. Included studies are only from seven countries due to the non-availability of studies from the remaining four countries. This constraint may affect the generalisation of the result to represent the sub-region. Likewise, fewer studies were available in some of the groups during subgroup analyses which may affect the estimates in the respective groups. Thirdly, only HEV seroprevalence was considered in this analysis; sporadic HEV infection and epidemics that equally contributes to the disease burden were not estimated.

## Conclusion

Even though there are no reports from some countries in the sub-region, SEA has high HEV seroprevalence. As expected, the prevalence is higher in some countries than others, and the variation is attributable to detectable, concealed, and random factors. Therefore, there is a need for concerted efforts towards preventing and controlling this emerging disease at national and regional levels. Increased research, surveillance, and purposeful screening of at-risk groups and blood donors will assist in prevention and control.

## Supplementary Information

Supplementary Information 1.

Supplementary Information 2.

Supplementary Information 3.

Supplementary Figure 1.

Supplementary Figure 2.

Supplementary Figure 3.

Supplementary Figure 4.

## Data Availability

All data generated or analysed during this study are included in this published article and its Supplementary Information files.
