# Supplementary material for: Meta-analysis and moderator analysis of the seroprevalence of hepatitis E in South-Eastern Asia
Source: Sci Rep. 2023 Jul 23;13:11880. doi: 10.1038/s41598-023-37941-0 (PMC10363542; doi:10.1038/s41598-023-37941-0)
Supplement: Supplementary file 2 — Supplementary Information 2. [file 41598_2023_37941_MOESM2_ESM.docx]

**S2 File**

**Eligibility criteria from the study protocol**

- Inclusion criteria
  - - Study location; studies conducted in any of the South East Asian countries. South East Asia countries as used by the United Nations (United Nations Statistics Division).
    - Context; the study will include all observational studies (cross – sectional studies, intervention studies, cohort studies, longitudinal prevalence studies, seroprevalence studies, prevalence surveys) that report the prevalence (seroprevalence) of HEV infection in any of the study locations.
    - Time period; no limitations placed on the year of publication.
    - Language of publication; English.
    - Participants; Susceptible groups and non – susceptible groups (healthy general population, healthy blood donors, urban residents).
    - Age; any age range.
- Exclusion criteria
  - - Studies that did not clearly separate the prevalence of HEV infection from other viral diseases and studies conducted in animals only.
    - Sporadic infection and epidemic studies of hepatitis E were excluded.
    - Studies conducted in non – southeast Asian population.
    - Studies with suboptimal methodology and studies covering topics other than HEV epidemiology (laboratory studies on pathogenesis of diseases, molecular biology).
    - Studies without clear description of population, serological assay, time period and place of sample collection.
    - Case reports, letters, books, dissertations, review articles, unpublished reports and conference papers.

**Search strategy:**

The search strategy will embrace the assessment of all relevant literature citations captured through the application of the search algorithm in five electronic bibliographic databases. Also, a grey literature search will be conducted via hand searching references of selected (review) articles and conference proceedings. Additionally, a corresponding internet search will be done in Google scholar, Google and Yahoo applying the same algorithm used in the bibliographic database search.

**Databases**

The five Selected databases to be used include Scopus, Science Direct, PubMed, MEDLINE and ASEAN Citation Index. The electronic search strategy will be as follows:

**Library/Database:** Scopus

**Date of Search:** March 22, 2020

**Search String:**  Advanced search ((“Hepatitis E Virus” OR “HEV infection” OR “HEV” OR “Viral Hepatitis E” OR “Hepatitis E” OR “Hepatitis E virus infection” OR “Hepatitis E antibodies”) AND (Seroepidem* OR “Prevalence” OR Epidem* OR “Survey” OR “Seroprevalence”) (“Indonesia” OR “Vietnam” OR “Thailand” OR “Singapore” OR “Malaysia” OR “Philippines” OR “Cambodia” OR “Myanmar” OR “Burma” OR “Laos” OR “Brunei” OR “Timor-Leste”))

**# Hits:2,438**

**Library/Database:** Science Direct

**Date of Search:** March 22, 2020

**Search String:**  Articles (“Hepatitis E Virus” OR “HEV infection” OR “HEV” OR “Viral Hepatitis E” OR “Hepatitis E” OR “Hepatitis E virus infection” OR “Hepatitis E antibodies”) AND (Seroepidem* OR “Prevalence” OR Epidem* OR “Survey” OR “Seroprevalence”)

Title, Abstract, or Author – specified Keywords (“Indonesia” OR “Vietnam” OR “Thailand” OR “Singapore” OR “Malaysia” OR “Philippines” OR “Cambodia” OR “Myanmar” OR “Burma” OR “Laos” OR “Brunei” OR “Timor-Leste”)

**# Hits: 51**

**Library/Database:** MEDLINE

**Date of Search:** March 22, 2020

**Search String:** All fields ((“Hepatitis E Virus” OR “HEV infection” OR “HEV” OR “Viral Hepatitis E” OR “Hepatitis E” OR “Hepatitis E virus infection” OR “Hepatitis E antibodies”) AND (Seroepidem* OR “Prevalence” OR Epidem* OR “Survey” OR “Seroprevalence”) AND (“Indonesia” OR “Vietnam” OR “Thailand” OR “Singapore” OR “Malaysia” OR “Philippines” OR “Cambodia” OR “Myanmar” OR “Burma” OR “Laos” OR “Brunei” OR “Timor-Leste”))

**# Hits: 1,478**

**Database:** PubMed

**Date of Search:** March 22, 2020

**Search String: (**(“Hepatitis E Virus” OR “HEV infection” OR “HEV” OR “Viral Hepatitis E” OR “Hepatitis E” OR “Hepatitis E virus infection” OR “Hepatitis E antibodies”) AND (Seroepidem* OR “Prevalence” OR Epidem* OR “Survey” OR “Seroprevalence”) AND (“Indonesia” OR “Vietnam” OR “Thailand” OR “Singapore” OR “Malaysia” OR “Philippines” OR “Cambodia” OR “Myanmar” OR “Burma” OR “Laos” OR “Brunei” OR “Timor-Leste”))

**# Hits: 184**

**Database:** ASEAN Citation Index

**Date of Search:** March 22, 2020

**Search String:** All fields ((“Hepatitis E Virus” OR “HEV infection” OR “HEV” OR “Viral Hepatitis E” OR “Hepatitis E” OR “Hepatitis E virus infection” OR “Hepatitis E antibodies”) AND (Seroepidem* OR “Prevalence” OR Epidem* OR “Survey” OR “Seroprevalence”) AND (“Indonesia” OR “Vietnam” OR “Thailand” OR “Singapore” OR “Malaysia” OR “Philippines” OR “Cambodia” OR “Myanmar” OR “Burma” OR “Laos” OR “Brunei” OR “Timor-Leste”))

**# Hits: 0**

**# Hits:**

Total # of citations prior to de-duplication: 4,151

Total # of citations after 1^st^ round of de-duplication in Excel: 3,368

Total # citations added from manual search: 6

**Quality assessment**

The appraisal tool used for the included observational studies presented below as extracted from the Munn et al (2014) study (Munn et al., 2014).

Prevalence Critical Appraisal Instrument

The 10 criteria used to assess the methodological quality of studies reporting prevalence data and an explanation are described below. These questions can be answered either with a yes, no, unclear, or not applicable.

Answers: Yes, No, Unclear or Not/Applicable

1. Was the sample representative of the target population?

This question relies upon knowledge of the broader characteristics of the population of interest. If the study is of women with breast cancer, knowledge of at least the characteristics, demographics, and medical history is needed. The term “target population” should not be taken to infer every individual from everywhere or with similar disease or exposure characteristics. Instead, give consideration to specific population characteristics in the study, including age range, gender, morbidities, medications, and other potentially influential factors. For example, a sample may not be representative of the target population if a certain group has been used (such as those working for one organisation, or one profession) and the results then inferred to the target population (i.e. working adults).

2. Were study participants recruited in an appropriate way?

Recruitment is the calling or advertising strategy for gaining interest in the study, and is not the same as sampling. Studies may report random sampling from a population, and the methods section should report how sampling was performed. What source of data were study participants recruited from? Was the sampling frame appropriate? For example, census data is a good example of appropriate recruitment as a good census will identify everybody. Was everybody included who should have been included? Were any groups of persons excluded? Was the whole population of interest surveyed? If not, was random sampling from a defined subset of the population employed? Was stratified random sampling with eligibility criteria used to ensure the sample was representative of the population that the researchers were generalizing to?

3. Was the sample size adequate?

An adequate sample size is important to ensure good precision of the final estimate. Ideally, we are looking for evidence that the authors conducted a sample size calculation to determine an adequate sample size. This will estimate how many subjects are needed to produce a reliable estimate of the measure(s) of interest. For conditions with a low prevalence, a larger sample size is needed. Also consider sample sizes for subgroup (or characteristics) analyses, and whether these are appropriate. Sometimes, the study will be large enough (as in large national surveys) whereby a sample size calculation is not required. In these cases, sample size can be considered adequate.

When there is no sample size calculation and it is not a large national survey, the reviewers may consider conducting their own sample size analysis using the following formula (24,25):

n=Z2P(1−P)d2

Where:

n= sample size

Z= Z statistic for a level of confidence

P= Expected prevalence or proportion (in proportion of one; if 20%, P= 0.2)

d= precision (in proportion of one; if 5%, d= 0.05)

4. Were the study subjects and setting described in detail?

Certain diseases or conditions vary in prevalence across different geographic regions and populations (e.g. women vs. men, socio-demographic variables between countries). Has the study sample been described in sufficient detail so that other researchers can determine if it is comparable to the population of interest to them?

5. Is the data analysis conducted with sufficient coverage of the identified sample?

A large number of dropouts, refusals or “not founds” amongst selected subjects may diminish a study’s validity, as can low response rates for survey studies.

- Did the authors describe the reasons for non-response and compare persons in the study to those not in the study, particularly with regards to their socio-demographic characteristics?

- Could the not-responders have led to an underestimate of prevalence of the disease or condition under investigation?

- If reasons for non-response appear to be unrelated to the outcome measured and the characteristics of non-responders are comparable to those in the study, the researchers may be able to justify a more modest response rate.

- Did the means of assessment or measurement negatively affect the response rate (measurement should be easily accessible, conveniently timed for participants, acceptable in length, and suitable in content).

6. Were objective, standard criteria used for measurement of the condition?

Here we are looking for measurement or classification bias. Many health problems are not easily diagnosed or defined and some measures may not be capable of including or excluding appropriate levels or stages of the health problem. If the outcomes were assessed based on existing definitions or diagnostic criteria, then the answer to this question is likely to be yes. If the outcomes were assessed using observer reported, or self-reported scales, the risk of over- or under-reporting is increased, and objectivity is compromised. Importantly, determine if the measurement tools used were validated instruments as this has a significant impact on outcome assessment validity.

7. Was the condition measured reliably?

Considerable judgment is required to determine the presence of some health outcomes. Having established the objectivity of the outcome measurement instrument (see item 6 of this scale), it is important to establish how the measurement was conducted. Were those involved in collecting data trained or educated in the use of the instrument/s? If there was more than one data collector, were they similar in terms of level of education, clinical or research experience, or level of responsibility in the piece of research being appraised? - Has the researcher justified the methods chosen? - Has the researcher made the methods explicit? (For interview method, how were interviews conducted?)

8. Was there appropriate statistical analysis?

As with any consideration of statistical analysis, consideration should be given to whether there was a more appropriate alternate statistical method that could have been used. The methods section should be detailed enough for reviewers to identify the analytical technique used and how specific variables were measured. Additionally, it is also important to assess the appropriateness of the analytical strategy in terms of the assumptions associated with the approach as differing methods of analysis are based on differing assumptions about the data and how it will respond. Prevalence rates found in studies only provide estimates of the true prevalence of a problem in the larger population. Since some subgroups are very small, 95% confidence intervals are usually given.

9. Are all important confounding factors/ subgroups/differences identified and accounted for?

Incidence and prevalence studies often draw or report findings regarding the differences between groups. It is important that authors of these studies identify all important confounding factors, subgroups and differences and account for these.

10. Were subpopulations identified using objective criteria?

Objective criteria should also be used where possible to identify subgroups (refer to question 6).

**Summary result for study quality assessment of the included studies**

| **S/N** | **Study ID** | **Study type** | **Decision** | **Quality score** |
| --- | --- | --- | --- | --- |
| 1 | (Hinjoy et al., 2013) | Seroprevalence study | Included | 100% |
| 2 | (Sa-nguanmoo et al., 2015) | Seroprevalence study | Included | 80% |
| 3 | (Poovorawan et al., 1996) | Seroprevalence study | Included | 70% |
| 4 | (Pilakasiri et al., 2009) | Seroprevalence study | Included | 100% |
| 5 | (Jupattanasin et al., 2019) | Seroprevalence study | Included | 100% |
| 6 | (Khounvisith et al., 2018) | Seroprevalence study | Included | 100% |
| 7 | (Tritz et al., 2018) | Seroprevalence study | Included | 100% |
| 8 | (Chow et al., 1996) | Seroprevalence study | Included | 80% |
| 9 | (Wong et al., 2019) | Seroprevalence study | Included | 78% |
| 10 | (Tran et al., 2003) | Seroprevalence study | Included | 70% |
| 11 | (Hau et al., 1999) | Seroprevalence study | Included | 100% |
| 12 | (Hoan et al., 2019) | Seroprevalence study | Included | 100% |
| 13 | (Hoan et al., 2015) | Seroprevalence study | Included | 100% |
| 14 | (Berto et al., 2018) | Seroprevalence study | Included | 100% |
| 15 | (Nouhin et al., 2015) | Seroprevalence study | Included | 100% |
| 16 | (Yamada et al., 2015) | Seroprevalence study | Included | 100% |
| 17 | (Nouhin et al., 2016) | Seroprevalence study | Included | 80% |
| 18 | (Nouhin et al., 2019) | Seroprevalence study | Included | 100% |
| 19 | (Ng et al., 2000) | Seroprevalence study | Included | 90% |
| 20 | (Seow et al., 1999) | Seroprevalence study | Included | 80% |
| 21 | (Hudu et al., 2018) | Seroprevalence study | Included | 90% |
| 22 | (Surya et al., 2005) | Seroprevalence study | Included | 90% |
| 23 | (Utsumi et al., 2011) | Seroprevalence study | Included | 90% |
| 24 | (Wibawa et al., 2004) | Seroprevalence study | Included | 100% |
| 25 | (Widasari et al., 2013) | Seroprevalence study | Included | 90% |
| 26 | (Achwan et al., 2007) | Seroprevalence study | Included | 80% |
| 27 | (Gonwong et al., 2014) | Seroprevalence study | Included | 100% |
| 28 | (Holt et al., 2016) | Seroprevalence study | Included | 100% |
| 29 | (Corwin et al., 1995) | Seroprevalence study | Included | 90% |
| 30 | (Corwin et al., 1996) | Seroprevalence study | Included | 80% |
| 31 | (Wibawa et al., 2004) | Seroprevalence study | Included | 100% |
| 32 | (Wibawa et al., 2007) | Seroprevalence study | Included | 80% |
| 33 | (Sedyaningsih-Mamahit et al., 2002) | Sporadic infection | Excluded | 89% |
| 34 | (Uchida et al., 1993) | Outbreak study | Excluded | 89% |
| 835 | (Gloriani-Barzaga et al., 1997) | Sporadic infection | Excluded | 90% |

**References**

ACHWAN, W. A., MUTTAQIN, Z., ZAKARIA, E., DEPAMEDE, S. A., SUMOHARJO, S., TSUDA, F., TAKAHASHI, K., ABE, N. & MISHIRO, S. 2007. Epidemiology of hepatitis B, C, and E viruses and human immunodeficiency virus infections in Tahuna, Sangihe-Talaud Archipelago, Indonesia. *Intervirology,* 50**,** 408-411.

BERTO, A., PHAM, H., THAO, T., VY, N., CADDY, S., HIRAIDE, R., TUE, N., GOODFELLOW, I., CARRIQUE‐MAS, J. & THWAITES, G. 2018. Hepatitis E in southern Vietnam: Seroepidemiology in humans and molecular epidemiology in pigs. *Zoonoses and public health,* 65**,** 43-50.

CHOW, W., NG, H., LIM, G. & OON, C. 1996. Hepatitis E in Singapore-a seroprevalence study. *Singapore medical journal,* 37**,** 579-582.

CORWIN, A., DAI, T., DUC, D. D., SUU, P., VAN, N. T., HA, L. D., JANICK, M., KANTI, L., SIE, A. & SODERQUIST, R. 1996. Acute viral hepatitis in Hanoi, Viet Nam. *Transactions of the Royal Society of Tropical Medicine and Hygiene,* 90**,** 647-648.

CORWIN, A., JAROT, K., LUBIS, I., NASUTION, K., SUPARMAWO, S., SUMARDIATI, A., WIDODO, S., NAZIR, S., ORNDORFF, G. & CHOI, Y. 1995. Two years' investigation of epidemic hepatitis E virus transmission in West Kalimantan (Borneo), Indonesia. *Transactions of the Royal Society of Tropical Medicine and Hygiene,* 89**,** 262-265.

GLORIANI-BARZAGA, N., CABANBAN, A., GRAHAM, R. R. & FLORESE, R. H. 1997. Hepatitis E virus infection diagnosed by serology: a report of cases at the San Lazaro Hospital, Manila. *Phil J Microbiol Infect Dis,* 26**,** 169-172.

GONWONG, S., CHUENCHITRA, T., KHANTAPURA, P., ISLAM, D., SIRISOPANA, N. & MASON, C. J. 2014. Pork consumption and seroprevalence of hepatitis E virus,Thailand, 2007-2008. *Emerging infectious diseases,* 20**,** 1531-1534.

HAU, C. H., HIEN, T. T., TIEN, N., KHIEM, H. B., SAC, P. K., NHUNG, V. T., LARASATI, R. P., LARAS, K., PUTRI, M. P. & DOSS, R. 1999. Prevalence of enteric hepatitis A and E viruses in the Mekong River delta region of Vietnam. *The American journal of tropical medicine and hygiene,* 60**,** 277-280.

HINJOY, S., NELSON, K. E., GIBBONS, R., JARMAN, R., MONGKOLSIRICHAIKUL, D., SMITHSUWAN, P., FERNANDEZ, S., LABRIQUE, A. B. & PATCHANEE, P. 2013. A cross‐sectional study of hepatitis E virus infection in healthy people directly exposed and unexposed to pigs in a rural community in northern Thailand. *Zoonoses and public health,* 60**,** 555-562.

HOAN, N. X., HUY, P. X., SY, B. T., MEYER, C. G., SON, T. V., BINH, M. T., GIANG, D. P., TU ANH, D., BOCK, C.-T. & WANG, B. High Hepatitis E virus (HEV) Positivity Among Domestic Pigs and Risk of HEV Infection of Individuals Occupationally Exposed to Pigs and Pork Meat in Hanoi, Vietnam. Open forum infectious diseases, 2019. Oxford University Press US, ofz306.

HOAN, N. X., VAN TONG, H., HECHT, N., SY, B. T., MARCINEK, P., MEYER, C. G., TOAN, N. L., KURRECK, J., KREMSNER, P. G. & BOCK, C.-T. 2015. Hepatitis E virus superinfection and clinical progression in hepatitis B patients. *EBioMedicine,* 2**,** 2080-2086.

HOLT, H. R., INTHAVONG, P., KHAMLOME, B., BLASZAK, K., KEOKAMPHE, C., SOMOULAY, V., PHONGMANY, A., DURR, P. A., GRAHAM, K. & ALLEN, J. 2016. Endemicity of zoonotic diseases in pigs and humans in lowland and upland Lao PDR: identification of socio-cultural risk factors. *PLoS neglected tropical diseases,* 10**,** e0003913.

HUDU, S. A., NIAZLIN, M. T., NORDIN, S. A., HARMAL, N. S., TAN, S. S., OMAR, H., SHAHAR, H., MUTALIB, N. A. & SEKAWI, Z. 2018. Hepatitis E virus isolated from chronic hepatitis B patients in Malaysia: Sequences analysis and genetic diversity suggest zoonotic origin. *Alexandria journal of medicine,* 54**,** 487-494.

JUPATTANASIN, S., CHAINUVATI, S., CHOTIYAPUTTA, W., CHANMANEE, T., SUPAPUENG, O., CHAROONRUANGRIT, U., OOTA, S. & LOUISIRIROTCHANAKUL, S. 2019. A nationwide survey of the seroprevalence of hepatitis E virus infections among blood donors in Thailand. *Viral Immunology,* 32**,** 302-307.

KHOUNVISITH, V., TRITZ, S., KHENKHA, L., PHOUTANA, V., KEOSENGTHONG, A., POMMASICHAN, S., NOUANTHONG, P., HÜBSCHEN, J. M., SNOECK, C. J. & REINHARZ, D. 2018. High circulation of Hepatitis E virus in pigs and professionals exposed to pigs in Laos. *Zoonoses and public health,* 65**,** 1020-1026.

MUNN, Z., MOOLA, S., RIITANO, D. & LISY, K. 2014. The development of a critical appraisal tool for use in systematic reviews addressing questions of prevalence. *International journal of health policy and management,* 3**,** 123-128.

NG, K., HE, J., SAW, T. & LYLES, C. 2000. A seroprevalence study of viral hepatitis E infection in human immunodeficiency virus type 1 infected subjects in Malaysia. *The Medical journal of Malaysia,* 55**,** 58-64.

NOUHIN, J., BARENNES, H., MADEC, Y., PRAK, S., HOU, S. V., KERLEGUER, A., KIM, S., PEAN, P. & ROUET, F. 2015. Low frequency of acute hepatitis E virus (HEV) infections but high past HEV exposure in subjects from Cambodia with mild liver enzyme elevations, unexplained fever or immunodeficiency due to HIV-1 infection. *Journal of Clinical Virology,* 71**,** 22-27.

NOUHIN, J., MADEC, Y., PRAK, S., ORK, M., KERLEGUER, A., FROEHLICH, Y., PAVIO, N. & ROUET, F. 2019. Declining hepatitis E virus antibody prevalence in Phnom Penh, Cambodia during 1996–2017. *Epidemiology & Infection,* 147.

NOUHIN, J., PRAK, S., MADEC, Y., BARENNES, H., WEISSEL, R., HOK, K., PAVIO, N. & ROUET, F. 2016. Hepatitis E virus antibody prevalence, RNA frequency, and genotype among blood donors in Cambodia (Southeast Asia). *Transfusion,* 56**,** 2597-2601.

PILAKASIRI, C., GIBBONS, R. V., JARMAN, R. G., SUPYAPOUNG, S. & MYINT, K. S. A. 2009. Hepatitis antibody profile of Royal Thai Army nursing students. *Tropical Medicine & International Health,* 14**,** 609-611.

POOVORAWAN, Y., THEAMBOONLERS, A., CHUMDERMPADETSUK, S., KOMOLMIT, P. & THONG, C. 1996. Prevalence of hepatitis E virus infection in Thailand. *Annals of Tropical Medicine & Parasitology,* 90**,** 189-196.

SA-NGUANMOO, P., POSUWAN, N., VICHAIWATTANA, P., WUTTHIRATKOWIT, N., OWATANAPANICH, S., WASITTHANKASEM, R., THONGMEE, T., POOVORAWAN, K., THEAMBOONLERS, A. & VONGPUNSAWAD, S. 2015. Swine is a possible source of hepatitis E virus infection by comparative study of hepatitis A and E seroprevalence in Thailand. *PloS one,* 10.

SEDYANINGSIH-MAMAHIT, E., LARASATI, R., LARAS, K., SIDEMEN, A., SUKRI, N., SABARUDDIN, N., DIDI, S., SARAGIH, J., MYINT, K. & ENDY, T. 2002. First documented outbreak of hepatitis E virus transmission in Java, Indonesia. *Transactions of the Royal Society of Tropical Medicine and Hygiene,* 96**,** 398-404.

SEOW, H. F., MAHOMED, N. M. B., MAK, J. W., RIDDELL, M. A., LI, F. & ANDERSON, D. A. 1999. Seroprevalence of antibodies to hepatitis E virus in the normal blood donor population and two aboriginal communities in Malaysia. *Journal of medical virology,* 59**,** 164-168.

SURYA, I. G. P., KORNIA, K., SUWARDEWA, T. G. A., TSUDA, F. & MISHIRO, S. 2005. Serological markers of hepatitis B, C, and E viruses and human immunodeficiency virus type‐1 infections in pregnant women in Bali, Indonesia. *Journal of medical virology,* 75**,** 499-503.

TRAN, H. T.-T., USHIJIMA, H., QUANG, V. X., PHUONG, N., LI, T.-C., HAYASHI, S., LIEN, T. X., SATA, T. & ABE, K. 2003. Prevalence of hepatitis virus types B through E and genotypic distribution of HBV and HCV in Ho Chi Minh City, Vietnam. *Hepatology research,* 26**,** 275-280.

TRITZ, S. E., KHOUNVISITH, V., POMMASICHAN, S., NINNASOPHA, K., KEOSENGTHONG, A., PHOUTANA, V., CAMOIN, M., HÜBSCHEN, J. M., BLACK, A. P. & MULLER, C. P. 2018. Evidence of increased Hepatitis E virus exposure in Lao villagers with contact to ruminants. *Zoonoses and public health,* 65**,** 690-701.

UCHIDA, T., AYE, T. T., MA, X., IIDA, F., SHIKATA, T., ICHIKAWA, M., RIKIHISA, T. & WIN, K. M. 1993. An epidemic outbreak of hepatitis E in Yangon of Myanmar: antibody assay and animal transmission of the virus. *Pathology International,* 43**,** 94-98.

UTSUMI, T., HAYASHI, Y., LUSIDA, M. I., AMIN, M., HENDRA, A., YANO, Y. & HOTTA, H. 2011. Prevalence of hepatitis E virus among swine and humans in two different ethnic communities in Indonesia. *Archives of virology,* 156**,** 689-693.

WIBAWA, I. D. N., MULJONO, D. H., SURYADARMA, I., TSUDA, F., TAKAHASHI, M., NISHIZAWA, T. & OKAMOTO, H. 2004. Prevalence of antibodies to hepatitis E virus among apparently healthy humans and pigs in Bali, Indonesia: Identification of a pig infected with a genotype 4 hepatitis E virus. *Journal of medical virology,* 73**,** 38-44.

WIBAWA, I. D. N., SURYADARMA, I., TSUDA, F., MATSUMOTO, Y., NINOMIYA, M., TAKAHASHI, M. & OKAMOTO, H. 2007. Identification of genotype 4 hepatitis E virus strains from a patient with acute hepatitis E and farm pigs in Bali, Indonesia. *Journal of medical virology,* 79**,** 1138-1146.

WIDASARI, D. I., YANO, Y., UTSUMI, T., HERIYANTO, D. S., ANGGOROWATI, N., RINONCE, H. T., UTORO, T., LUSIDA, M. I., ASMARA, W. & HOTTA, H. 2013. Hepatitis E virus infection in two different regions of Indonesia with identification of swine HEV genotype 3. *Microbiology and immunology,* 57**,** 692-703.

WONG, C. C., THEAN, S. M., NG, Y., KANG, J. S. L., NG, T. Y., CHAU, M. L., KOH, T. H. & CHAN, K. P. 2019. Seroepidemiology and genotyping of hepatitis E virus in Singapore reveal rise in number of cases and similarity of human strains to those detected in pig livers. *Zoonoses and public health,* 66**,** 773-782.

YAMADA, H., TAKAHASHI, K., LIM, O., SVAY, S., CHUON, C., HOK, S., DO, S. H., FUJIMOTO, M., AKITA, T. & GOTO, N. 2015. Hepatitis E virus in Cambodia: Prevalence among the general population and complete genome sequence of genotype 4. *PLoS One,* 10**,** e0136903.
