# Supplementary material for: Meta-analysis and moderator analysis of the seroprevalence of hepatitis E in South-Eastern Asia
Source: Sci Rep. 2023 Jul 23;13:11880. doi: 10.1038/s41598-023-37941-0 (PMC10363542; doi:10.1038/s41598-023-37941-0)
Supplement: Supplementary file 3 — Supplementary Information 3. [file 41598_2023_37941_MOESM3_ESM.docx]

**S3 File**

**Item 1: pooled prevalence**

>m.prop<-metaprop(event = Case, n = Sample_size, studlab = Study_ID, data = DataSet1, method = "GLMM", sm = "PLOGIT", fixed = FALSE, random = TRUE, hakn = TRUE, title = "PoolPrevalence")

> summary(m.prop)

**Review: PoolPrevalence**

proportion 95%-CI

Nouhin 2019 0.4112 [0.3895; 0.4331]

Nouhin 2016 0.2824 [0.2322; 0.3369]

Yamada 2015 0.1843 [0.1591; 0.2118]

Nouhin 2015 0.3006 [0.2695; 0.3332]

Wibawa 2004a 0.0655 [0.0517; 0.0816]

Wibawa 2004b 0.1807 [0.1546; 0.2092]

Utsumi 2011 0.0988 [0.0650; 0.1424]

Surya 2005 0.1844 [0.1584; 0.2127]

Wibawa 2007 0.4035 [0.2756; 0.5418]

Corwin 1995 0.5865 [0.5392; 0.6327]

Widasari 2013 0.0776 [0.0555; 0.1049]

Achwan 2007 0.0585 [0.0409; 0.0808]

Tritz et 2018 0.5184 [0.4627; 0.5738]

Khounvisith 2018 0.2722 [0.2262; 0.3221]

Hudu 2018 0.0976 [0.0431; 0.1832]

Seow 1999 0.2672 [0.2115; 0.3291]

Ng 2000 0.1448 [0.0919; 0.2128]

Wong 2019 0.2229 [0.2088; 0.2376]

Chow 1996 0.1233 [0.0828; 0.1743]

Sa-nguanmoo 2015 0.3689 [0.3336; 0.4053]

Pilakasiri 2009 0.1155 [0.0852; 0.1519]

Hinjoy 2013 0.2300 [0.1943; 0.2689]

Jupattanasin 2019 0.2968 [0.2614; 0.3342]

Poovorawan 1996 0.0678 [0.0522; 0.0862]

Gonwong 2014 0.1399 [0.1323; 0.1479]

Siripanyaphinyo 2014 0.3869 [0.3459; 0.4291]

Hoan 2019 0.4479 [0.4014; 0.4951]

Hoan 2015 0.4168 [0.3929; 0.4409]

Berto 2018 0.2955 [0.2756; 0.3160]

Hau 1999 0.0898 [0.0689; 0.1145]

Tran 2003 0.4216 [0.3495; 0.4963]

Corwin 1996 0.0933 [0.0659; 0.1274]

Number of studies combined: k = 32

Number of observations: o = 29944

Number of events: e = 6806

proportion 95%-CI

Random effects model 0.2138 [0.1658; 0.2711]

Quantifying heterogeneity:

tau^2 = 0.7341; tau = 0.8568; I^2 = 98.7% [98.5%; 98.8%]; H = 8.68 [8.09; 9.31]

Test of heterogeneity:

Q d.f. p-value Test

2336.27 31 0 Wald-type

2630.19 31 0 Likelihood-Ratio

Details on meta-analytical method:

- Random intercept logistic regression model

- Maximum-likelihood estimator for tau^2

- Hartung-Knapp adjustment for random effects model

- Logit transformation

- Clopper-Pearson confidence interval for individual studies

**Item 2: Outliers**

>find.outliers(m.prop)

Identified outliers (random-effects model)

------------------------------------------

"Nouhin 2019", "Wibawa 2004a", "Utsumi 2011", "Wibawa 2007", "Corwin 1995", "Widasari 2013", "Achwan 2007", "Tritz et 2018", "Sa-nguanmoo 2015", "Pilakasiri 2009", "Poovorawan 1996", "Gonwong 2014", "Siripanyaphinyo 2014", "Hoan 2019", "Hoan 2015", "Berto 2018", "Hau 1999", "Tran 2003", "Corwin 1996"

Results with outliers removed

-----------------------------

Review: PoolPrevalence

Number of studies combined: k = 13

Number of observations: o = 29944

Number of events: e = 6806

proportion 95%-CI

Random effects model 0.2134 [0.1782; 0.2533]

Quantifying heterogeneity:

tau^2 = 0.1156; tau = 0.3400; I^2 = 88.8% [82.6%; 92.7%]; H = 2.98 [2.40; 3.71]

Test of heterogeneity:

Q d.f. p-value Test

106.68 12 < 0.0001 Wald-type

110.89 12 < 0.0001 Likelihood-Ratio

Details on meta-analytical method:

- Random intercept logistic regression model

- Maximum-likelihood estimator for tau^2

- Hartung-Knapp adjustment for random effects model

- Logit transformation

**Item 3: Influence Analysis**

>m.rma

Random-Effects Model (k = 32; tau^2 estimator: ML)

tau^2 (estimated amount of total heterogeneity): 0.7258 (SE = 0.1866)

tau (square root of estimated tau^2 value): 0.8520

I^2 (total heterogeneity / total variability): 99.03%

H^2 (total variability / sampling variability): 103.58

Test for Heterogeneity:

Q(df = 31) = 2336.2674, p-val< .0001

Model Results:

estimate se tvaldfpval ci.lb ci.ub ​

-1.2957 0.1554 -8.3395 31 <.0001 -1.6126 -0.9789 ***

---

Signif. codes: 0 ‘***’ 0.001 ‘**’ 0.01 ‘*’ 0.05 ‘.’ 0.1 ‘ ’ 1

**Item 4: GOSH**

>res.gosh.diag

GOSH Diagnostics

================================

- Number of K-means clusters detected: 2

- Number of DBSCAN clusters detected: 5

- Number of GMM clusters detected: 4

Identification of potential outliers

---------------------------------

- K-means: Study 10, Study 1, Study 13

- DBSCAN: Study 25, Study 1, Study 8, Study 10, Study 18, Study 23, Study 19, Study 28

- Gaussian Mixture Model: Study 25, Study 1, Study 8, Study 10, Study 18, Study 23, Study 19, Study 28>

**Item 5: Removal of Identified Outliers**

>update.meta(m.prop, exclude = c(25, 1, 8, 10, 18, 23, 19, 28, 13))%>%

+ summary()

Review: PoolPrevalence

proportion 95%-CI exclude

Nouhin 2019 0.4112 [0.3895; 0.4331] *

Nouhin 2016 0.2824 [0.2322; 0.3369]

Yamada 2015 0.1843 [0.1591; 0.2118]

Nouhin 2015 0.3006 [0.2695; 0.3332]

Wibawa 2004a 0.0655 [0.0517; 0.0816]

Wibawa 2004b 0.1807 [0.1546; 0.2092]

Utsumi 2011 0.0988 [0.0650; 0.1424]

Surya 2005 0.1844 [0.1584; 0.2127] *

Wibawa 2007 0.4035 [0.2756; 0.5418]

Corwin 1995 0.5865 [0.5392; 0.6327] *

Widasari 2013 0.0776 [0.0555; 0.1049]

Achwan 2007 0.0585 [0.0409; 0.0808]

Tritz et 2018 0.5184 [0.4627; 0.5738] *

Khounvisith 2018 0.2722 [0.2262; 0.3221]

Hudu 2018 0.0976 [0.0431; 0.1832]

Seow 1999 0.2672 [0.2115; 0.3291]

Ng 2000 0.1448 [0.0919; 0.2128]

Wong 2019 0.2229 [0.2088; 0.2376] *

Chow 1996 0.1233 [0.0828; 0.1743] *

Sa-nguanmoo 2015 0.3689 [0.3336; 0.4053]

Pilakasiri 2009 0.1155 [0.0852; 0.1519]

Hinjoy 2013 0.2300 [0.1943; 0.2689]

Jupattanasin 2019 0.2968 [0.2614; 0.3342] *

Poovorawan 1996 0.0678 [0.0522; 0.0862]

Gonwong 2014 0.1399 [0.1323; 0.1479] *

Siripanyaphinyo 2014 0.3869 [0.3459; 0.4291]

Hoan 2019 0.4479 [0.4014; 0.4951]

Hoan 2015 0.4168 [0.3929; 0.4409] *

Berto 2018 0.2955 [0.2756; 0.3160]

Hau 1999 0.0898 [0.0689; 0.1145]

Tran 2003 0.4216 [0.3495; 0.4963]

Corwin 1996 0.0933 [0.0659; 0.1274]

Number of studies combined: k = 23

Number of observations: o = 29944

Number of events: e = 6806

proportion 95%-CI

Random effects model 0.1850 [0.1366; 0.2457]

Quantifying heterogeneity:

tau^2 = 0.6724; tau = 0.8200; I^2 = 97.7% [97.2%; 98.1%]; H = 6.59 [5.97; 7.28]

Test of heterogeneity:

Q d.f. p-value Test

956.33 22 < 0.0001 Wald-type

1164.42 22 < 0.0001 Likelihood-Ratio

Details on meta-analytical method:

- Random intercept logistic regression model

- Maximum-likelihood estimator for tau^2

- Hartung-Knapp adjustment for random effects model

- Logit transformation

- Clopper-Pearson confidence interval for individual studies

**Item 6: Subgroup Analysis**

**Country subgroup**

>update.meta(m.prop, subgroup = Country, tau.common = FALSE)

Review: PoolPrevalence

Number of studies combined: k = 32

Number of observations: o = 29944

Number of events: e = 6806

proportion 95%-CI

Random effects model 0.2138 [0.1658; 0.2711]

Quantifying heterogeneity:

tau^2 = 0.7341; tau = 0.8568; I^2 = 98.7% [98.5%; 98.8%]; H = 8.68 [8.09; 9.31]

Test of heterogeneity:

Q d.f. p-value Test

2336.27 31 0 Wald-type

2630.19 31 0 Likelihood-Ratio

Results for subgroups (random effects model):

k proportion 95%-CI tau^2 tau Q I^2

Country = Cambodia 4 0.2886 [0.1748; 0.4371] 0.1588 0.3985 143.55 97.9%

Country = Indonesia 8 0.1605 [0.0731; 0.3169] 1.0966 1.0472 570.84 98.8%

Country = Laos 2 0.3883 [0.0054; 0.9867] 0.2677 0.5174 41.78 97.6%

Country = Malaysia 3 0.1666 [0.0520; 0.4211] 0.2005 0.4477 14.04 85.8%

Country = Singapore 2 0.1743 [0.0082; 0.8433] 0.1060 0.3256 11.55 91.3%

Country = Thailand 7 0.2041 [0.1130; 0.3405] 0.5620 0.7497 532.66 98.9%

Country = Vietnam 6 0.2593 [0.1197; 0.4739] 0.7971 0.8928 315.16 98.4%

Test for subgroup differences (random effects model):

Q d.f. p-value

Between groups 11.81 6 0.0662

Details on meta-analytical method:

- Random intercept logistic regression model

- Maximum-likelihood estimator for tau^2

- Hartung-Knapp adjustment for random effects model

- Logit transformation

**Sampling Duration**

>update.meta(m.prop, subgroup = SamplingDuration, tau.common = FALSE)

Review: PoolPrevalence

Number of studies combined: k = 32

Number of observations: o = 29944

Number of events: e = 6806

proportion 95%-CI

Random effects model 0.2138 [0.1658; 0.2711]

Quantifying heterogeneity:

tau^2 = 0.7341; tau = 0.8568; I^2 = 98.7% [98.5%; 98.8%]; H = 8.68 [8.09; 9.31]

Test of heterogeneity:

Q d.f. p-value Test

2336.27 31 0 Wald-type

2630.19 31 0 Likelihood-Ratio

Results for subgroups (random effects model):

k proportion 95%-CI tau^2 tau

SamplingDuration = 10 and above 1 0.4112 [0.3898; 0.4329] -- --

SamplingDuration = 1 to 4 29 0.2054 [0.1554; 0.2663] 0.7737 0.8796

SamplingDuration = 5 to 9 2 0.2571 [0.0592; 0.6558] 0.0339 0.1843

Q I^2

SamplingDuration = 10 and above -- --

SamplingDuration = 1 to 4 1999.83 98.6%

SamplingDuration = 5 to 9 34.62 97.1%

Test for subgroup differences (random effects model):

Q d.f. p-value

Between groups 53.53 2 < 0.0001

Details on meta-analytical method:

- Random intercept logistic regression model

- Maximum-likelihood estimator for tau^2

- Hartung-Knapp adjustment for random effects model

- Logit transformation

**Diagnostic Method**

>update.meta(m.prop, subgroup = Diagnostic_Method, tau.common = FALSE)

Review: PoolPrevalence

Number of studies combined: k = 32

Number of observations: o = 29944

Number of events: e = 6806

proportion 95%-CI

Random effects model 0.2138 [0.1658; 0.2711]

Quantifying heterogeneity:

tau^2 = 0.7341; tau = 0.8568; I^2 = 98.7% [98.5%; 98.8%]; H = 8.68 [8.09; 9.31]

Test of heterogeneity:

Q d.f. p-value Test

2336.27 31 0 Wald-type

2630.19 31 0 Likelihood-Ratio

Results for subgroups (random effects model):

k proportion 95%-CI tau^2 tau Q

Diagnostic_Method = Total Ig 11 0.2696 [0.1748; 0.3914] 0.6583 0.8114 427.49

Diagnostic_Method = IgG 21 0.1885 [0.1366; 0.2544] 0.6935 0.8328 1299.21

I^2

Diagnostic_Method = Total Ig 97.7%

Diagnostic_Method = IgG 98.5%

Test for subgroup differences (random effects model):

Q d.f. p-value

Between groups 2.23 1 0.1350

Details on meta-analytical method:

- Random intercept logistic regression model

- Maximum-likelihood estimator for tau^2

- Hartung-Knapp adjustment for random effects model

- Logit transformation

**Assay type used**

>update.meta(m.prop, subgroup = Assay_type_used, tau.common = FALSE)

Review: PoolPrevalence

Number of studies combined: k = 32

Number of observations: o = 29944

Number of events: e = 6806

proportion 95%-CI

Random effects model 0.2138 [0.1658; 0.2711]

Quantifying heterogeneity:

tau^2 = 0.7341; tau = 0.8568; I^2 = 98.7% [98.5%; 98.8%]; H = 8.68 [8.09; 9.31]

Test of heterogeneity:

Q d.f. p-value Test

2336.27 31 0 Wald-type

2630.19 31 0 Likelihood-Ratio

Results for subgroups (random effects model):

k proportion 95%-CI tau^2

Assay_type_used = Wantai Bio-Pharm 5 0.2743 [0.1636; 0.4223] 0.2504

Assay_type_used = Others 4 0.2399 [0.0776; 0.5422] 0.6695

Assay_type_used = Mizuo et al., method 4 0.1762 [0.0582; 0.4254] 0.5822

Assay_type_used = Genelabs Diagnostics 3 0.1958 [0.0099; 0.8559] 1.6305

Assay_type_used = MP Biomedicals 4 0.2570 [0.0762; 0.5920] 0.8013

Assay_type_used = In-house assay 2 0.1749 [0.0000; 0.9999] 1.5030

Assay_type_used = Euroimmum, Lubeck 3 0.3141 [0.2214; 0.4245] 0.0275

Assay_type_used = Abbott Laboratory 3 0.1248 [0.0264; 0.2849] --

Assay_type_used = WRAIR EIA 2 0.1665 [0.0047; 0.8936] 0.1549

Assay_type_used = DIA.PRO Diagnostic 2 0.2417 [0.0007; 0.9929] 0.4544

tau Q I^2

Assay_type_used = Wantai Bio-Pharm 0.5004 90.28 95.6%

Assay_type_used = Others 0.8182 157.77 98.1%

Assay_type_used = Mizuo et al., method 0.7630 95.94 96.9%

Assay_type_used = Genelabs Diagnostics 1.2769 362.94 99.4%

Assay_type_used = MP Biomedicals 0.8952 334.69 99.1%

Assay_type_used = In-house assay 1.2260 113.49 99.1%

Assay_type_used = Euroimmum, Lubeck 0.1658 13.00 84.6%

Assay_type_used = Abbott Laboratory -- -- --

Assay_type_used = WRAIR EIA 0.3936 18.67 94.6%

Assay_type_used = DIA.PRO Diagnostic 0.6741 209.56 99.5%

Test for subgroup differences (random effects model):

Q d.f. p-value

Between groups 104.99 10 < 0.0001

Details on meta-analytical method:

- Random intercept logistic regression model

- Maximum-likelihood estimator for tau^2

- Hartung-Knapp adjustment for random effects model

- Logit transformation

**Location**

>update.meta(m.prop, subgroup = Location, tau.common = FALSE)

Review: PoolPrevalence

Number of studies combined: k = 32

Number of observations: o = 29944

Number of events: e = 6806

proportion 95%-CI

Random effects model 0.2138 [0.1658; 0.2711]

Quantifying heterogeneity:

tau^2 = 0.7341; tau = 0.8568; I^2 = 98.7% [98.5%; 98.8%]; H = 8.68 [8.09; 9.31]

Test of heterogeneity:

Q d.f. p-value Test

2336.27 31 0 Wald-type

2630.19 31 0 Likelihood-Ratio

Results for subgroups (random effects model):

k proportion 95%-CI tau^2 tau Q I^2

Location = Urban 21 0.1989 [0.1428; 0.2700] 0.7456 0.8635 1242.84 98.4%

Location = Rural 9 0.2366 [0.1331; 0.3848] 0.8151 0.9028 734.82 98.9%

Location = Mixed 2 0.2889 [0.1352; 0.5135] 0 0 0.72 0.0%

Test for subgroup differences (random effects model):

Q d.f. p-value

Between groups 6.17 2 0.0456

Details on meta-analytical method:

- Random intercept logistic regression model

- Maximum-likelihood estimator for tau^2

- Hartung-Knapp adjustment for random effects model

- Logit transformation

**Studied population**

>update.meta(m.prop, subgroup = Studied_population, tau.common = FALSE)

Review: PoolPrevalence

Number of studies combined: k = 32

Number of observations: o = 29944

Number of events: e = 6806

proportion 95%-CI

Random effects model 0.2138 [0.1658; 0.2711]

Quantifying heterogeneity:

tau^2 = 0.7341; tau = 0.8568; I^2 = 98.7% [98.5%; 98.8%]; H = 8.68 [8.09; 9.31]

Test of heterogeneity:

Q d.f. p-value Test

2336.27 31 0 Wald-type

2630.19 31 0 Likelihood-Ratio

Results for subgroups (random effects model):

k proportion 95%-CI tau^2 tau Q

Studied_population = Healthy 18 0.1836 [0.1300; 0.2528] 0.6596 0.8122 1371.19

Studied_population = Clinical 10 0.2015 [0.1257; 0.3070] 0.5815 0.7626 265.60

Studied_population = Mixed 4 0.4323 [0.2765; 0.6028] 0.1820 0.4267 153.79

I^2

Studied_population = Healthy 98.8%

Studied_population = Clinical 96.6%

Studied_population = Mixed 98.0%

Test for subgroup differences (random effects model):

Q d.f. p-value

Between groups 19.82 2 < 0.0001

Details on meta-analytical method:

- Random intercept logistic regression model

- Maximum-likelihood estimator for tau^2

- Hartung-Knapp adjustment for random effects model

- Logit transformation

**Year of Publication**

>update.meta(m.prop, subgroup = PublicationYear, tau.common = FALSE)

Review: HEVPooledPrevalence

Number of studies combined: k = 32

Number of observations: o = 29944

Number of events: e = 6806

proportion 95%-CI

Random effects model 0.2138 [0.1658; 0.2711]

Quantifying heterogeneity:

tau^2 = 0.7341; tau = 0.8568; I^2 = 98.7% [98.5%; 98.8%]; H = 8.68 [8.09; 9.31]

Test of heterogeneity:

Q d.f. p-value Test

2336.27 31 0 Wald-type

2630.19 31 0 Likelihood-Ratio

Results for subgroups (random effects model):

k proportion 95%-CI tau^2 tau Q

PublicationYear = 2015-2019 13 0.3093 [0.2463; 0.3803] 0.2581 0.5080 480.49

PublicationYear = 2000-2004 4 0.1718 [0.0512; 0.4435] 0.6906 0.8310 152.06

PublicationYear = 2010-2014 5 0.1642 [0.0735; 0.3273] 0.5154 0.7179 258.29

PublicationYear = 2005-2009 4 0.1546 [0.0451; 0.4146] 0.6888 0.8299 70.64

PublicationYear = =1999 6 0.1623 [0.0613; 0.3649] 1.0504 1.0249 490.98

I^2

PublicationYear = 2015-2019 97.5%

PublicationYear = 2000-2004 98.0%

PublicationYear = 2010-2014 98.5%

PublicationYear = 2005-2009 95.8%

PublicationYear = =1999 99.0%

Test for subgroup differences (random effects model):

Q d.f. p-value

Between groups 11.69 4 0.0198

Details on meta-analytical method:

- Random intercept logistic regression model

- Maximum-likelihood estimator for tau^2

- Hartung-Knapp adjustment for random effects model

- Logit transformation

**Mean Age Interval**

>update.meta(m.prop, subgroup = MeanAge, tau.common = FALSE)

Review: HEVPooledPrevalence

Number of studies combined: k = 32

Number of observations: o = 29944

Number of events: e = 6806

proportion 95%-CI

Random effects model 0.2138 [0.1658; 0.2711]

Quantifying heterogeneity:

tau^2 = 0.7341; tau = 0.8568; I^2 = 98.7% [98.5%; 98.8%]; H = 8.68 [8.09; 9.31]

Test of heterogeneity:

Q d.f. p-value Test

2336.27 31 0 Wald-type

2630.19 31 0 Likelihood-Ratio

Results for subgroups (random effects model):

k proportion 95%-CI tau^2 tau Q I^2

MeanAge = 32-37 5 0.2300 [0.0966; 0.4548] 0.6748 0.8215 402.34 99.0%

MeanAge = 26-31 9 0.2096 [0.1161; 0.3487] 0.8124 0.9013 486.87 98.4%

MeanAge = 38-43 4 0.1849 [0.0706; 0.4038] 0.4495 0.6704 96.76 96.9%

MeanAge = =50 5 0.1343 [0.0664; 0.2529] 0.3539 0.5949 87.28 95.4%

MeanAge = 44-49 4 0.4385 [0.3675; 0.5121] 0.0267 0.1633 16.12 81.4%

MeanAge = 20-25 5 0.1815 [0.0820; 0.3549] 0.5178 0.7196 141.85 97.2%

Test for subgroup differences (random effects model):

Q d.f. p-value

Between groups 57.25 5 < 0.0001

Details on meta-analytical method:

- Random intercept logistic regression model

- Maximum-likelihood estimator for tau^2

- Hartung-Knapp adjustment for random effects model

- Logit transformation

**Gender**

>update.meta(m.prop, subgroup = Gender, tau.common = FALSE)

Review: HEVPooledPrevalence

Number of studies combined: k = 32

Number of observations: o = 29944

Number of events: e = 6806

proportion 95%-CI

Random effects model 0.2138 [0.1658; 0.2711]

Quantifying heterogeneity:

tau^2 = 0.7341; tau = 0.8568; I^2 = 98.7% [98.5%; 98.8%]; H = 8.68 [8.09; 9.31]

Test of heterogeneity:

Q d.f. p-value Test

2336.27 31 0 Wald-type

2630.19 31 0 Likelihood-Ratio

Results for subgroups (random effects model):

k proportion 95%-CI tau^2 tau Q I^2

Gender = Male 20 0.2330 [0.1711; 0.3090] 0.6599 0.8123 1678.31 98.9%

Gender = Female 12 0.1848 [0.1132; 0.2872] 0.8015 0.8952 648.27 98.3%

Test for subgroup differences (random effects model):

Q d.f. p-value

Between groups 0.83 1 0.3609

Details on meta-analytical method:

- Random intercept logistic regression model

- Maximum-likelihood estimator for tau^2

- Hartung-Knapp adjustment for random effects model

- Logit transformation

**Sample Size**

>update.meta(m.prop, subgroup = SampleSize, tau.common = FALSE)

Review: HEVPooledPrevalence

Number of studies combined: k = 32

Number of observations: o = 29944

Number of events: e = 6806

proportion 95%-CI

Random effects model 0.2138 [0.1658; 0.2711]

Quantifying heterogeneity:

tau^2 = 0.7341; tau = 0.8568; I^2 = 98.7% [98.5%; 98.8%]; H = 8.68 [8.09; 9.31]

Test of heterogeneity:

Q d.f. p-value Test

2336.27 31 0 Wald-type

2630.19 31 0 Likelihood-Ratio

Results for subgroups (random effects model):

k proportion 95%-CI tau^2 tau Q I^2

SampleSize = >2000 4 0.2546 [0.1276; 0.4437] 0.2822 0.5312 742.97 99.6%

SampleSize = >100-500 13 0.2299 [0.1444; 0.3455] 0.8644 0.9298 579.59 97.9%

SampleSize = >500-1000 11 0.1884 [0.1220; 0.2794] 0.5719 0.7562 474.46 97.9%

SampleSize = >1000-2000 2 0.1831 [0.0000; 0.9999] 1.3468 1.1605 314.68 99.7%

SampleSize = =100 2 0.2138 [0.0001; 0.9992] 0.7691 0.8770 15.91 93.7%

Test for subgroup differences (random effects model):

Q d.f. p-value

Between groups 1.35 4 0.8521

Details on meta-analytical method:

- Random intercept logistic regression model

- Maximum-likelihood estimator for tau^2

- Hartung-Knapp adjustment for random effects model

- Logit transformation

**Item 6: Egger’s test**

>metabias(m.prop, method.bias = "linreg")

Review: HEVPooledPrevalence

Linear regression test of funnel plot asymmetry

Test result: t = -0.68, df = 30, p-value = 0.5038

Sample estimates:

bias se.bias intercept se.intercept

-2.0246 2.9917 -0.9525 0.2445

Details:

- multiplicative residual heterogeneity variance (tau^2 = 76.7046)

- predictor: standard error

- weight: inverse variance

- reference: Egger et al. (1997), BMJ

>eggers.test(m.prop)

Eggers' test of the intercept

=============================

intercept 95% CI t p

-2.025 -7.89 - 3.84 -0.677 0.5037612

Eggers' test does not indicate the presence of funnel plot asymmetry.

**Item 7: Meta-regression Meta-analysis**

pred ci.lbci.ub pi.lb pi.ub

0.2148 0.1679 0.2706 0.0465 0.6055

>print(pes.logit, digits=4)

Random-Effects Model (k = 32; tau^2 estimator: REML)

tau^2 (estimated amount of total heterogeneity): 0.7500 (SE = 0.1958)

tau (square root of estimated tau^2 value): 0.8661

I^2 (total heterogeneity / total variability): 99.07%

H^2 (total variability / sampling variability): 107.00

Test for Heterogeneity:

Q(df = 31) = 2336.2674, p-val< .0001

Model Results:

estimate se zvalpval ci.lb ci.ub ​

-1.2960 0.1552 -8.3492 <.0001 -1.6003 -0.9918 ***

---

Signif. codes: 0 ‘***’ 0.001 ‘**’ 0.01 ‘*’ 0.05 ‘.’ 0.1 ‘ ’ 1

>confint(pes.logit, digits=2)

estimate ci.lb ci.ub

tau^2 0.75 0.48 1.35

tau 0.87 0.69 1.16

I^2(%) 99.07 98.53 99.48

H^2 107.00 68.15 191.22

**Item 7a: Univariate Meta-regression**

**Location**

Mixed-Effects Model (k = 32; tau^2 estimator: REML)

tau^2 (estimated amount of residual heterogeneity): 0.7851 (SE = 0.2117)

tau (square root of estimated tau^2 value): 0.8861

I^2 (residual heterogeneity / unaccounted variability): 99.02%

H^2 (unaccounted variability / sampling variability): 102.53

R^2 (amount of heterogeneity accounted for): 0.00%

Test for Residual Heterogeneity:

QE(df = 29) = 1978.3792, p-val< .0001

Test of Moderators (coefficients 2:3):

QM(df = 2) = 0.7256, p-val = 0.6957

Model Results:

estimate se zvalpval ci.lb ci.ub ​

intrcpt -0.9349 0.6324 -1.4783 0.1393 -2.1744 0.3046

LocationRural -0.2323 0.6996 -0.3320 0.7399 -1.6034 1.1389

LocationUrban -0.4518 0.6621 -0.6824 0.4950 -1.7495 0.8459

---

Signif. codes: 0 ‘***’ 0.001 ‘**’ 0.01 ‘*’ 0.05 ‘.’ 0.1 ‘ ’ 1

**Diagnostic Method**

Mixed-Effects Model (k = 32; tau^2 estimator: REML)

tau^2 (estimated amount of residual heterogeneity): 0.7199 (SE = 0.1912)

tau (square root of estimated tau^2 value): 0.8485

I^2 (residual heterogeneity / unaccounted variability): 98.98%

H^2 (unaccounted variability / sampling variability): 97.61

R^2 (amount of heterogeneity accounted for): 4.01%

Test for Residual Heterogeneity:

QE(df = 30) = 1726.7032, p-val< .0001

Test of Moderators (coefficient 2):

QM(df = 1) = 2.0895, p-val = 0.1483

Model Results:

estimate se zvalpval ci.lb ci.ub ​

intrcpt -1.4542 0.1876 -7.7529 <.0001 -1.8218 -1.0866 ***

Diagnostic_MethodTotal Ig 0.4637 0.3208 1.4455 0.1483 -0.1650 1.0925

---

Signif. codes: 0 ‘***’ 0.001 ‘**’ 0.01 ‘*’ 0.05 ‘.’ 0.1 ‘ ’ 1

**Country**

Mixed-Effects Model (k = 32; tau^2 estimator: REML)

tau^2 (estimated amount of residual heterogeneity): 0.7774 (SE = 0.2256)

tau (square root of estimated tau^2 value): 0.8817

I^2 (residual heterogeneity / unaccounted variability): 98.85%

H^2 (unaccounted variability / sampling variability): 86.93

R^2 (amount of heterogeneity accounted for): 0.00%

Test for Residual Heterogeneity:

QE(df = 25) = 1629.5705, p-val< .0001

Test of Moderators (coefficients 2:7):

QM(df = 6) = 4.9596, p-val = 0.5490

Model Results:

estimate se zvalpval ci.lb ci.ub ​

intrcpt -0.9048 0.4431 -2.0419 0.0412 -1.7732 -0.0363 *

CountryIndonesia -0.7421 0.5448 -1.3622 0.1731 -1.8100 0.3257

CountryLaos0.4506 0.7692 0.5858 0.5580 -1.0571 1.9582

CountryMalaysia -0.7385 0.6918 -1.0675 0.2857 -2.0944 0.6174

CountrySingapore -0.6914 0.7719 -0.8957 0.3704 -2.2042 0.8215

CountryThailand -0.4542 0.5558 -0.8172 0.4138 -1.5436 0.6352

CountryVietnam -0.1410 0.5730 -0.2461 0.8056 -1.2640 0.9819

---

Signif. codes: 0 ‘***’ 0.001 ‘**’ 0.01 ‘*’ 0.05 ‘.’ 0.1 ‘ ’ 1

**Assay type Used**

Mixed-Effects Model (k = 32; tau^2 estimator: REML)

tau^2 (estimated amount of residual heterogeneity): 0.9069 (SE = 0.2862)

tau (square root of estimated tau^2 value): 0.9523

I^2 (residual heterogeneity / unaccounted variability): 99.09%

H^2 (unaccounted variability / sampling variability): 110.25

R^2 (amount of heterogeneity accounted for): 0.00%

Test for Residual Heterogeneity:

QE(df = 21) = 1396.3804, p-val< .0001

Test of Moderators (coefficients 2:11):

QM(df = 10) = 4.6406, p-val = 0.9139

Model Results:

estimate se zvalpval ci.lb​

intrcpt -2.2951 0.6827 -3.3619 0.0008 -3.6331

Assay_type_usedAbbott Laboratory 0.5193 1.1953 0.4345 0.6639 -1.8233

Assay_type_usedDIA.PRO Diagnostic 1.1545 0.9600 1.2025 0.2292 -0.7272

Assay_type_usedEuroimmum, Lubeck 1.5015 0.8783 1.7095 0.0874 -0.2200

Assay_type_usedGenelabs Diagnostics 0.8941 0.8809 1.0150 0.3101 -0.8324

Assay_type_usedIn-house assay 0.7540 0.9658 0.7806 0.4350 -1.1391

Assay_type_usedMizuo et al., method 0.7663 0.8361 0.9164 0.3594 -0.8725

Assay_type_usedMP Biomedicals 1.2359 0.8339 1.4821 0.1383 -0.3985

Assay_type_usedOthers1.1450 0.8355 1.3704 0.1706 -0.4926

Assay_type_usedWantai Bio-Pharm 1.2803 0.8084 1.5838 0.1132 -0.3041

Assay_type_usedWRAIR EIA 0.6763 0.9637 0.7018 0.4828 -1.2124

ci.ub

intrcpt -0.9571 ***

Assay_type_usedAbbott Laboratory 2.8620

Assay_type_usedDIA.PRO Diagnostic 3.0362

Assay_type_usedEuroimmum, Lubeck 3.2230 .

Assay_type_usedGenelabs Diagnostics 2.6206

Assay_type_usedIn-house assay 2.6470

Assay_type_usedMizuo et al., method 2.4050

Assay_type_usedMP Biomedicals 2.8703

Assay_type_usedOthers 2.7827

Assay_type_usedWantai Bio-Pharm 2.8647

Assay_type_usedWRAIR EIA 2.5651

---

Signif. codes: 0 ‘***’ 0.001 ‘**’ 0.01 ‘*’ 0.05 ‘.’ 0.1 ‘ ’ 1

**Studied population**

Mixed-Effects Model (k = 32; tau^2 estimator: REML)

tau^2 (estimated amount of residual heterogeneity): 0.6284 (SE = 0.1705)

tau (square root of estimated tau^2 value): 0.7927

I^2 (residual heterogeneity / unaccounted variability): 98.75%

H^2 (unaccounted variability / sampling variability): 79.75

R^2 (amount of heterogeneity accounted for): 16.22%

Test for Residual Heterogeneity:

QE(df = 29) = 1790.5790, p-val< .0001

Test of Moderators (coefficients 2:3):

QM(df = 2) = 7.7603, p-val = 0.0206

Model Results:

estimate se zvalpval ci.lb ci.ub ​

intrcpt -1.3669 0.2581 -5.2967 <.0001 -1.8727 -0.8611 ***

Studied_populationHealthy -0.1200 0.3199 -0.3751 0.7076 -0.7471 0.5071

Studied_populationMixed1.0989 0.4745 2.3160 0.0206 0.1689 2.0289 *

---

Signif. codes: 0 ‘***’ 0.001 ‘**’ 0.01 ‘*’ 0.05 ‘.’ 0.1 ‘ ’ 1

**Gender**

Mixed-Effects Model (k = 32; tau^2 estimator: REML)

tau^2 (estimated amount of residual heterogeneity): 0.7541 (SE = 0.2001)

tau (square root of estimated tau^2 value): 0.8684

I^2 (residual heterogeneity / unaccounted variability): 99.06%

H^2 (unaccounted variability / sampling variability): 106.60

R^2 (amount of heterogeneity accounted for): 0.00%

Test for Residual Heterogeneity:

QE(df = 30) = 2326.5798, p-val< .0001

Test of Moderators (coefficient 2):

QM(df = 1) = 0.8226, p-val = 0.3644

Model Results:

estimate se zvalpval ci.lb ci.ub ​

intrcpt -1.4777 0.2536 -5.8267 <.0001 -1.9747 -0.9806 ***

GenderMale0.2913 0.3212 0.9070 0.3644 -0.3382 0.9209

---

Signif. codes: 0 ‘***’ 0.001 ‘**’ 0.01 ‘*’ 0.05 ‘.’ 0.1 ‘ ’ 1

**Sample Size**

Mixed-Effects Model (k = 32; tau^2 estimator: REML)

tau^2 (estimated amount of residual heterogeneity): 0.7737 (SE = 0.2053)

tau (square root of estimated tau^2 value): 0.8796

I^2 (residual heterogeneity / unaccounted variability): 98.96%

H^2 (unaccounted variability / sampling variability): 95.73

R^2 (amount of heterogeneity accounted for): 0.00%

Test for Residual Heterogeneity:

QE(df = 30) = 1803.6345, p-val< .0001

Test of Moderators (coefficient 2):

QM(df = 1) = 0.0844, p-val = 0.7715

Model Results:

estimate se zvalpval ci.lb ci.ub ​

intrcpt -1.2654 0.1902 -6.6512 <.0001 -1.6382 -0.8925 ***

Sample_size -0.0000 0.0001 -0.2904 0.7715 -0.0003 0.0002

---

Signif. codes: 0 ‘***’ 0.001 ‘**’ 0.01 ‘*’ 0.05 ‘.’ 0.1 ‘ ’ 1

**Year of publication**

Mixed-Effects Model (k = 32; tau^2 estimator: REML)

tau^2 (estimated amount of residual heterogeneity): 0.6738 (SE = 0.1793)

tau (square root of estimated tau^2 value): 0.8208

I^2 (residual heterogeneity / unaccounted variability): 98.96%

H^2 (unaccounted variability / sampling variability): 95.87

R^2 (amount of heterogeneity accounted for): 10.17%

Test for Residual Heterogeneity:

QE(df = 30) = 2129.4578, p-val< .0001

Test of Moderators (coefficient 2):

QM(df = 1) = 4.2432, p-val = 0.0394

Model Results:

estimate se zvalpval ci.lb ci.ub ​

intrcpt -76.9335 36.7198 -2.0952 0.0362 -148.9029 -4.9640 *

Year_of_publication 0.0376 0.0183 2.0599 0.0394 0.0018 0.0734 *

---

Signif. codes: 0 ‘***’ 0.001 ‘**’ 0.01 ‘*’ 0.05 ‘.’ 0.1 ‘ ’ 1

**Duration of sampling year**

Mixed-Effects Model (k = 32; tau^2 estimator: REML)

tau^2 (estimated amount of residual heterogeneity): 0.7352 (SE = 0.1953)

tau (square root of estimated tau^2 value): 0.8574

I^2 (residual heterogeneity / unaccounted variability): 98.97%

H^2 (unaccounted variability / sampling variability): 97.16

R^2 (amount of heterogeneity accounted for): 1.98%

Test for Residual Heterogeneity:

QE(df = 30) = 2085.5668, p-val< .0001

Test of Moderators (coefficient 2):

QM(df = 1) = 1.6281, p-val = 0.2020

Model Results:

estimate se zvalpval ci.lb ci.ub ​

intrcpt -1.4270 0.1849 -7.7169 <.0001 -1.7894 -1.0646 ***

Duration_of_Sampling_year0.0525 0.0412 1.2760 0.2020 -0.0282 0.1333

---

Signif. codes: 0 ‘***’ 0.001 ‘**’ 0.01 ‘*’ 0.05 ‘.’ 0.1 ‘ ’ 1

**Mean age**

Mixed-Effects Model (k = 32; tau^2 estimator: REML)

tau^2 (estimated amount of residual heterogeneity): 0.7688 (SE = 0.2038)

tau (square root of estimated tau^2 value): 0.8768

I^2 (residual heterogeneity / unaccounted variability): 98.99%

H^2 (unaccounted variability / sampling variability): 99.31

R^2 (amount of heterogeneity accounted for): 0.00%

Test for Residual Heterogeneity:

QE(df = 30) = 1978.3183, p-val< .0001

Test of Moderators (coefficient 2):

QM(df = 1) = 0.2111, p-val = 0.6459

Model Results:

estimate se zvalpval ci.lb ci.ub ​

intrcpt -1.5393 0.5518 -2.7895 0.0053 -2.6208 -0.4578 **

Mean_Age0.0068 0.0149 0.4594 0.6459 -0.0223 0.0360

---

Signif. codes: 0 ‘***’ 0.001 ‘**’ 0.01 ‘*’ 0.05 ‘.’ 0.1 ‘ ’ 1

**Item 7b: Multivariate meta-regression**

> print(metareg.moderators)

Mixed-Effects Model (k = 32; tau^2 estimator: REML)

tau^2 (estimated amount of residual heterogeneity): 0.5805 (SE = 0.1669)

tau (square root of estimated tau^2 value): 0.7619

I^2 (residual heterogeneity / unaccounted variability): 98.40%

H^2 (unaccounted variability / sampling variability): 62.68

R^2 (amount of heterogeneity accounted for): 22.61%

Test for Residual Heterogeneity:

QE(df = 26) = 1188.3293, p-val< .0001

Test of Moderators (coefficients 2:6):

QM(df = 5) = 13.4809, p-val = 0.0193

Model Results:

estimate se zvalpval ci.lb ci.ub​

intrcpt -60.8813 39.4219 -1.5444 0.1225 -138.1468 16.3841

Duration_of_Sampling_year 0.0269 0.0395 0.6819 0.4953 -0.0504 0.1042

Year_of_publication 0.0296 0.0197 1.5042 0.1325 -0.0090 0.0682

Studied_populationHealthy -0.2283 0.3239 -0.7050 0.4808 -0.8631 0.4064

Studied_populationMixed 0.9347 0.4665 2.0036 0.0451 0.0204 1.8490

Diagnostic_MethodTotal Ig 0.1241 0.3183 0.3900 0.6966 -0.4998 0.7481

intrcpt

Duration_of_Sampling_year

Year_of_publication

Studied_populationHealthy

Studied_populationMixed *

Diagnostic_MethodTotal Ig

---

Signif. codes: 0 ‘***’ 0.001 ‘**’ 0.01 ‘*’ 0.05 ‘.’ 0.1 ‘ ’ 1
