# Supplementary material for: Meta-analysis and moderator analysis of the seroprevalence of hepatitis E in South-Eastern Asia
Source: Sci Rep. 2023 Jul 23;13:11880. doi: 10.1038/s41598-023-37941-0 (PMC10363542; doi:10.1038/s41598-023-37941-0)
Supplement: Supplementary file 5 — Supplementary Figure 2. [file 41598_2023_37941_MOESM5_ESM.docx]

Supplementary figure:


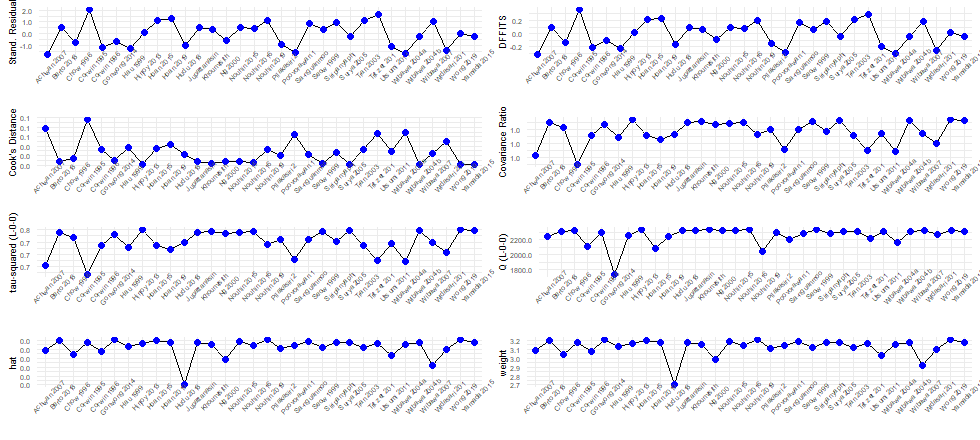


Figure 2: Influence diagnostics plot displays the values of different influence measures
